# Evolution and gene capture in ancient endogenous retroviruses - insights from the crocodilian genomes

**DOI:** 10.1186/s12977-014-0071-2

**Published:** 2014-12-12

**Authors:** Amanda Y Chong, Kenji K Kojima, Jerzy Jurka, David A Ray, Arian F A Smit, Sally R Isberg, Jaime Gongora

**Affiliations:** Faculty of Veterinary Science, University of Sydney, Sydney, NSW 2006 Australia; Genetic Information Research Institute, Los Altos, CA 94022 USA; Department of Biochemistry, Molecular Biology, Plant Pathology and Entomology, Mississippi State University, Starkville, Mississippi State 39762 USA; Institute for Genomics, Biocomputing and Biotechnology, Mississippi State University, Starkville, Mississippi State 39762 USA; Current Address: Department of Biological Sciences, Texas Tech University, Lubbock, TX 79409 USA; Institute for Systems Biology, Seattle, WA 98109-5234 USA; Centre for Crocodile Research, Noonamah, NT 0837 Australia

## Abstract

**Background:**

Crocodilians are thought to be hosts to a diverse and divergent complement of endogenous retroviruses (ERVs) but a comprehensive investigation is yet to be performed. The recent sequencing of three crocodilian genomes provides an opportunity for a more detailed and accurate representation of the ERV diversity that is present in these species. Here we investigate the diversity, distribution and evolution of ERVs from the genomes of three key crocodilian species, and outline the key processes driving crocodilian ERV proliferation and evolution.

**Results:**

ERVs and ERV related sequences make up less than 2% of crocodilian genomes. We recovered and described 45 ERV groups within the three crocodilian genomes, many of which are species specific. We have also revealed a new class of ERV, ERV4, which appears to be common to crocodilians and turtles, and currently has no characterised exogenous counterpart. For the first time, we formally describe the characteristics of this ERV class and its classification relative to other recognised ERV and retroviral classes. This class shares some sequence similarity and sequence characteristics with ERV3, although it is phylogenetically distinct from the other ERV classes. We have also identified two instances of gene capture by crocodilian ERVs, one of which, the capture of a host KIT-ligand mRNA has occurred without the loss of an ERV domain.

**Conclusions:**

This study indicates that crocodilian ERVs comprise a wide variety of lineages, many of which appear to reflect ancient infections. In particular, ERV4 appears to have a limited host range, with current data suggesting that it is confined to crocodilians and some lineages of turtles. Also of interest are two ERV groups that demonstrate evidence of host gene capture. This study provides a framework to facilitate further studies into non-mammalian vertebrates and highlights the need for further studies into such species.

**Electronic supplementary material:**

The online version of this article (doi:10.1186/s12977-014-0071-2) contains supplementary material, which is available to authorized users.

## Background

Endogenous retroviruses (ERVs) are one group of vertebrate transposable elements that replicate through an RNA intermediate. ERVs are unique in that they arise from germline infections by exogenous retroviruses. As such ERVs represent both endogenous mobile DNAs and the remnants of ancient infectious agents. Crocodilians have been shown to harbour a number of divergent ERV lineages that show little similarity to ERVs from other vertebrates. Until now, the characterisation of these crocodilian ERVs has focussed on fragments from the protease and reverse transcriptase (*pro-pol*) genes [1-4], or longer sequences recovered from a single species [5]. These methodologies are highly reliant on sequence conservation for recovery of ERV data, with the PCR surveys focussing on conserved domains, and therefore likely to have missed more divergent, degraded or rarer ERVs. This in turn may result in an underestimation of the true ERV complement of these species, limiting understanding of the impact that these elements may have had on genome evolution, and species biology.

The sequencing of three crocodilian genomes [6] provides the opportunity to further expand our knowledge and understanding of ERVs in these taxa, and obtain a more accurate representation of the ERV diversity that is present. The three sequenced species (*Alligator mississippiensis*, *Crocodylus porosus*, and *Gavialis gangeticus*) represent the three major taxonomic lineages present within the Order Crocodylia, namely the alligators, crocodiles, and gharials, respectively. Alligators and crocodiles diverged 97–103 million years ago (MYA), while the crocodile-gharial divergence is estimated to have occurred 47–49 MYA [7,8].

Preliminary estimates of the repetitive DNA content of these genomes suggest that upwards of 23.4% for all three species are made up of repetitive DNA [6]. Here we present a comprehensive study of ERVs from the genomes of these three key crocodilian species to establish the distribution, diversity, and evolution of ERVs in these species. Furthermore, characterisation of the complete proviral sequences of divergent ERV lineages will clarify the taxonomic position of these sequences relative to the recognised ERV classes and exogenous retroviral genera, and allow for a more detailed description of these elements. This study has the potential to shed light on the evolution and genome biology of reptiles, avians, and modern vertebrate taxa by allowing a better understanding of the diversity and divergence of the ERVs that may be present in these taxa. In addition, the sequence data and characteristics defined in this study will facilitate the discovery of novel ERVs and, potentially, the reconstruction of ancient ERV lineages.

ERVs and their exogenous counterparts are loosely grouped into three classes: Class I (ERV1, *Gammaretroviruses*, and *Epsilonretroviruses*), Class II (ERV2, *Alpharetroviruses*, *Betaretoviruses*, *Deltaretroviruses*, and *Lentiviruses*) and Class III (ERV3 and *Spumaviruses*) [9]. These classes can further be divided into ‘families’ or lineages which can be loosely defined as a group of related elements [10], likely to have originated from a single insertion or infection event. Nomenclature of these ERV groups is varied and depends largely on context and species. For example, human ERV groups are predominantly named as HERV or ERV, as in HERV-K and ERV3, and should not be confused with the broader ERV classes listed above. Similarly, the convention of designating ERV groups by species results in multiple similar terms for very different groups of ERVs. The use of the term CERV is one instance where it has been used to describe ERVs from *Pan troglodytes* (chimpanzee) as well as crocodilians [2,4,11], although alternative naming schemas for crocodilian ERVs (CrocERV, and the Repbase suffixes AMi/Ami, Crp, and Gav) are provided herein.

The replication, divergence, and sequence preservation of these ERV groups within a host genome is driven by a number of factors and mechanisms, including mode of replication, selective pressures, and consequent effects on genomic function [12-14]. The mode of replication also appears to dictate the extent of ERV proliferation in the host genomes [15], and may affect the evolutionary dynamics of these elements. The presence and completeness of retroviral genes and accessory domains can provide insights into the potential methods by which these ERVs may replicate within the host genome [12-16]. Previous studies have suggested that crocodilian ERVs may be capable of replication within the genome, as potentially intact ORFs have been found among those retroelements [4]. However, as these hypotheses were drawn from fragments of *pro-pol*, subsequent recovery of complete proviral insertions from the crocodilian genome sequences will provide the data required for more informed inferences about the replicative potential of these ERVs and the mechanisms by which this occurs in these species.

The number of copies in the genome can also be affected by the population structure [17] According to this model, repetitive elements are likely to be fixed in small subpopulations by genetic drift and eventually passed on to the surviving population. Under the neutral drift, the initial rate of fixation is the same as the rate of replication. One implication of this is that the presence of multiple families of ERVs reflects the presence of multiple subpopulations in the host population at the time of their origin.

ERVs are capable of incorporating host genes through recombination and incorporation of the host mRNA into the retroviral genome. This process requires transcription of the cellular gene along with proviral DNA, co-packaging of the chimeric RNA particle, followed by infection of a new cell and recombination of the chimeric RNA with the retroviral RNA genome prior to insertion of the recombinant proviral genome [18], and usually occurs at the expense of at least one retroviral domain [19]. Despite this, such captures may also have beneficial effects for the provirus, facilitating viral entry or replication. For example, the capture and incorporation of cellular proto-oncogenes into functional proviruses may stimulate host cell proliferation, providing naïve target cells for replication of these retroviruses [20].

To better establish the distribution and processes driving ERV evolution in crocodilians, we retrieved full length ERV insertions from the genome sequences of the three key crocodilian species described above, and classified these to determine the sequence characteristics, genomic structure, and distribution of each group within crocodilians. We formally describe a new class of ERV, and the phylogenetic relationship with other ERV classes. Here we present an overview of the diverse range of ERVs present in crocodilians, and offer insights into their evolution within the genomes of three key crocodilian species including an estimated integration time and relative levels of replication. We describe two instances of host gene capture involving a crocodilian KIT-ligand, and nectin3. We also explore the implications of our findings for theories of ERV evolution.

## Results

### Overview of recovered ERVs

The estimated ERV content of crocodilian genomes ranges from 1.22% in *G. gangeticus*, to 1.88% in *A. mississippiensis* [21]. The proportion of ERV chains detected by RetroTector is much less than this, making up between 0.14% and 0.26% of the crocodilian genomes, excluding solo LTRs and highly degraded ERV sequences. RetroTector recovered a total 2,056 retroelement chains with a minimum chain score of 300. Of these, 576 were treated as 'complete' as they had motifs from all three coding domains and both 5` and 3` LTRs present (Additional file 1: Table S1). The average length of ERV chains were 7,328 bases in *A. mississippiensis*, 7,175 in *C. porosus*, and 7,203 in *G. gangeticus*. An additional 339,610 solo LTRs were detected from the three genomes. The average length of solo LTRs ranged from 1473 to 1573 bases across the three species. However, due to a lack of distinguishing features within the LTRs of LTR retroelements [22], it is not possible to determine which of these are ERV related and which are derived from *Gypsy*-like insertions.

Additional screening for ERV related sequence using the Repbase detection pipeline generated a total of 187 ERV LTR consensus sequences and 109 consensus sequences from the internal portions of ERV insertions (Additional file 2). We successfully reconstructed entire RT domains without any frameshift or nonsense mutations for 80 of these ERVs. These consensus sequences are deposited in Repbase (http://www.girinst.org/repbase).

### Classification of crocodilian ERVs

We were able to classify 45 distinct CrocERV groups from the combined RetroTector and Repbase datasets. Using similarity to the repeat library of consensus sequences, a total of 40 ERV groups were defined from 295 ‘complete’ ERV sequences defined by RetroTector, ranging in size from 1 to 67 sequences (Additional file 3: Figure S1, Additional files 4 and 5). A further five groups encoding a pol protein were recovered that were not represented within the ‘complete’ ERV sequences defined above (CrocERV41–45) (Additional file 1: Table S2). Average amino acid similarities within these groups ranged from 0.39-0.85 (Additional file 1: Table S2).

The majority of ERV groups were lineage specific, with only twelve found in all three species. Eleven, four, and two families were found only in *A. mississippiensis*, *C. porosus*, and *G. gangeticus*, respectively. A further 16 were found in both *C. porosus* and *G. gangeticus* (classed as *Longirostres* as defined by Harshman et al. [23]). We identified six orthologous insertions between *C. porosus* and *G. gangeticus* (for the full details, see Additional file 4). Of these, five were from CrocERV1, and one was from CrocERV38. No orthologous insertions were identified between all three genomes.

The estimated ages of ERV groups based on proviruses that appeared to have two intact LTRs ranged from 0–221 million years (Additional file 1: Table S2), although not all defined groups could be dated in this way due to difficulty predicting LTR sequences of individual proviruses. Estimated insertion dates for each of these groups indicated that the detected ERVs represent integration events post crocodilian-avian divergence, with the majority of classified groups dating to around the alligator-crocodile divergence ~100MYA or later, For the most part, these dates corresponded with predicted divergence times for the major crocodilian lineages, although the estimated dates for some ERV groups suggested a much younger age than implied by their distributions among the crocodilian lineages. However, these dates should only be interpreted as rough approximations due to difficulties predicting and recovering individual LTRs by both methods implemented in this study.

The association of the defined ERV groups with previously described ERV sequences produced varying results. The *Gammaretrovirus*-like ERV1 lineage (previously named CERV1) [2,4] corresponds to CrocERV5, which appears to be present in all three crocodilian species. The *Epsilonretrovirus*-like lineage represented by haplotype 58 from Chong et al. [4] corresponds to CrocERV7 and appears to be specific to *C. porosus*. Interestingly, the ERV4 *pro-pol* fragments (previously CERV2) were more variable, with most of the ERV4 groups showing similarity to more than one of the *pro-pol* fragments. In particular the complete ERV sequences recovered from *C. niloticus* by Martin et al. [5] were most similar to CrocERV21.

### Interspecies comparisons

**Figure 1:**
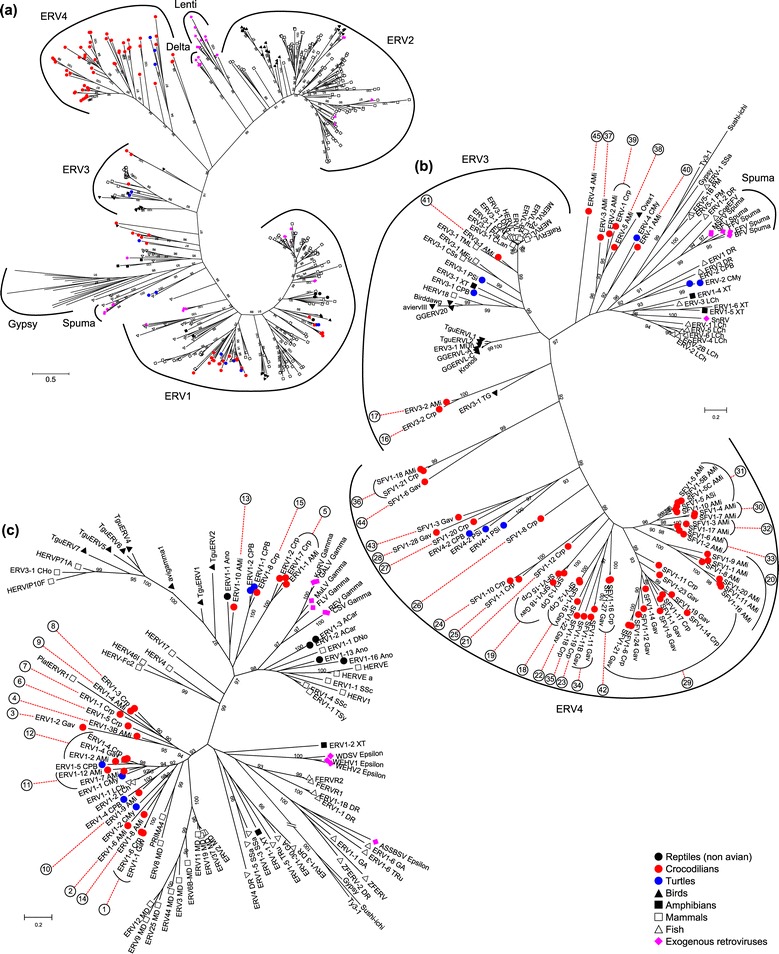
**Classification and likely relationships between the CrocERV groups and ERVs from other species.** Maximum likelihood phylogenies were created from the RT domain of the crocodilian consensus sequences and a selection of sequences deposited in Repbase and published sequences. Part **(a)** is the entire RT tree, while **(b)** and **(c)** are expanded versions of ERV3 and 4, and ERV1 respectively. The complete version of **(a)** including sequence IDs is presented as Additional file 6: Figure S2. Symbols represent the taxa from which the sequences were derived. The numbers of CrocERV groups are shown outside of corresponding consensus sequences. Major ERV and retroviral groups, and the *Gypsy* elements, are indicated by brackets. Numbers within the phylogeny indicate aLRT values greater than 90%. The scale bar indicates branch length.

**Figure 2:**
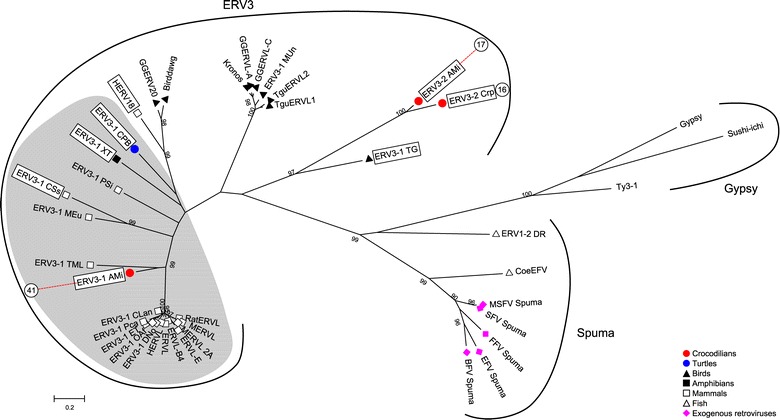
**Presence and absence of dUTPase and** ***env*** **is variable between lineages within ERV3.** Maximum likelihood phylogenies were created from the RT domain of Crocodilian ERV3 and ERV3 sequences deposited in Repbase. The ERV3 lineage encoding dUTPase is shaded in grey. Elements encoding a recognisable *env* are indicated by boxes. Symbols represent the taxa from which the sequences were derived. The numbers of CrocERV groups are shown outside of corresponding consensus sequences. Major retroviral groups are indicated brackets. Numbers within the phylogeny indicate aLRT values greater than 90%. The scale bar indicates branch length.

Phylogenetic clustering of ERV groups with exogenous and endogenous retroviruses from other species revealed that crocodilian ERVs clustered primarily with other reptilian ERV1 and ERV3 sequences, and within the newly defined ERV4 which is described in the following section (Figures 1 and 2). Llorens et al. [24] reported that retroviruses (exogenous and endogenous) could be classified into three classes (I, II and III), each corresponding to the expanded groupings for ERV1, 2, and 3. In our phylogeny, retroviral Class I (ERV1 and exogenous *Gamma-* and *Epsilonretroviruses*) and Class II (represented by exogenous *Alpha-*, *Beta-*, *Deltaretroviruses*, and *Lentiviruses*) are well supported. However, our phylogeny does not support a monophyletic grouping within Class III (*Spumaviruses* and ERV3), likely due to the diverse range of sequences included within the phylogeny. Although it is possible to use the term ERV3 for all ERVs that are neither ERV1 nor ERV2, we prefer to use the term ERV3 just for the monophyletic group including mammalian ERV3/ERVL elements. Instead, we introduce a term “ERV4” for the monophyletic group that includes the previously described crocodilian CERV2 as these ERVs are distinct from any other known retrovirus groups. The characteristics of ERV4 are described in the next section.

Based on the RT phylogeny, 15 CrocERV groups are classified as ERV1, 3 as ERV3, and 22 as ERV4. Five groups appeared to be intermediates between the major ERV classes and could not definitively be placed (Figure 1, Additional file 6: Figure S2, and Additional file 1: Table S2). With the exception of CrocERV41 and CrocERV45, crocodilian ERV families clustered with avian and reptilian ERV sequences.

A cluster including three CrocERVs (CrocERV5, 13, 15) and exogenous *Gammaretroviruses* is well supported by bootstrap analysis. Although statistical support is weak, the remaining crocodilian ERV1 families (CrocERV1-4, 6–12, 14) loosely cluster with groups from turtles (_CPB and _CMy), coelacanth (_LCh), primates (PRIMA4) and opossum (_MD). Overall similarity of the *pol* gene from each of the ERV classes was 0.43 for ERV1, 0.577 for ERV3, and 0.347 for ERV4.

Crocodilian ERV3 groups form two distinct clusters. The first of these, CrocERV41, is close to mammalian ERV3 and they share the presence of dUTPase domain downstream of integrase. The other group, which is composed by CrocERV16 and CrocERV17, are distant from mammalian ERV3 lineages. They are more related to avian ERV3 and lack a dUTPase domain. These three crocodilian groups as well as other ERV3 groups encode an envelope protein, indicating that the lack of *env* is not the shared feature of the whole ERV3 lineage (Figure 2).

Three CrocERVs (CrocERV37, 38, and 39) were clustered with the avian gene Ovex1 [25]. Sequence similarities were also observed between these groups and SpeV, a reported fragment of endogenous retrovirus from tuatara (NCBI: X85037) [26]. These avian and reptile ERV groups likely represent an ERV lineage that is absent in mammals. CrocERV45 is not clustered with any other retroviruses known to date. Although this group appears to sit close to Ovex1 (Figure 1) there is no phylogenetic support for an association between those branches. We could reconstruct envelope proteins for CrocERV37, 38, 39, 40 and 45, indicating that exogenous retroviruses related to these ERVs are or were present.

### Characteristics of ERV4

We have characterised the complete proviral structure of ERV4 groups from all three crocodilian genomes in addition to the related sequence fragments that have been recovered from 14 crocodilian species [2,4,5] (Chong et al., unpublished data). Related ERV groups were also recovered from two species of turtle, *Pelodiscus sinensis* (Chinese soft-shelled turtle; ERV4-1_PSi, ERV4-2_PSi, and ERV4-3_PSi; Kojima K.K. and Jurka, J., unpublished data) and *Chrysemys picta bellii* (painted turtle; ERV4-1_CPB and ERV4-2_CPB) [27], showing that ERV4 is not a crocodilian-specific group. We were unable to detect ERV4 sequences in other reptilian genomes, including *Chelonia mydas* (green sea turtle), *Anolis carolinensis* (green anole), and *Python bivittatus* (Burmese python), or in other vertebrates.

A number of sequence characteristics and domains can be used to help with the definition and distinction between ERV classes (Table 1). These include overall retroviral structure and the presence of additional accessory genes, zinc-fingers, a GPF/Y motif or equivalent, and the presence and location of dUTPase [9,24]. It should be noted that retroviruses and their exogenous counterparts are a heterogeneous family of viruses and repetitive elements, and therefore these traits may not be present in every member of these classes. The TSD length is another of the major characteristics to classify ERVs, although there is evidence to suggest that this is not consistent within the ERV classes (Additional file 1: Table S2). In general, ERV1 generates a 4 bp TSD, ERV2 generates a 6 bp TSD and ERV3 generates a 5 bp TSD [28].

**Table 1:** **Typical characteristics of ERV classes**

	**ERV1**	**ERV2**	**ERV3**	**ERV4**
TSD length	4 bp	6 bp	5 bp	5 bp
Zinc-finger motifs	1-2	2	Absent	Absent
GPF/Y motif or equivalent	Present	Present	Absent	Absent
dUTPase	Absent	Pro^ab^	Pol^b^	Absent

^a^Non-primate lentiviral ERVs encode dUTPase within *pol.*

^b^Some lineages may have lost dUTPase [9].

The domain structure of the ERV4 *pol* is consistent with other retroviruses; it includes an aspartyl protease called retropepsin, reverse transcriptase, ribonuclease H and DDE-type integrase. Structurally, ERV4 is very similar to ERV3. ERV4 lack zinc-finger motifs within *gag*, and a GPF/Y motif or sequence equivalent downstream of integrase. We were unable to detect the presence of dUTPase in any of the ERV4 groups. Unlike many ERV3 groups, ERV4 encodes a relatively intact *env*. However, this cannot be considered a distinguishing feature, as crocodilian ERV3 groups as well as those from a number of other species also encode *env* (Figure 2). Despite these structural similarities, our phylogenetic reconstructions of ERV phylogeny do not support a monophyletic grouping of ERV3 and ERV4 sequences. Therefore, at this stage, the classification of ERV4 must be based on the phylogenetic position of the *pol* protein.

### Capture of KIT-ligand mRNA by CrocERV29

**Figure 3:**
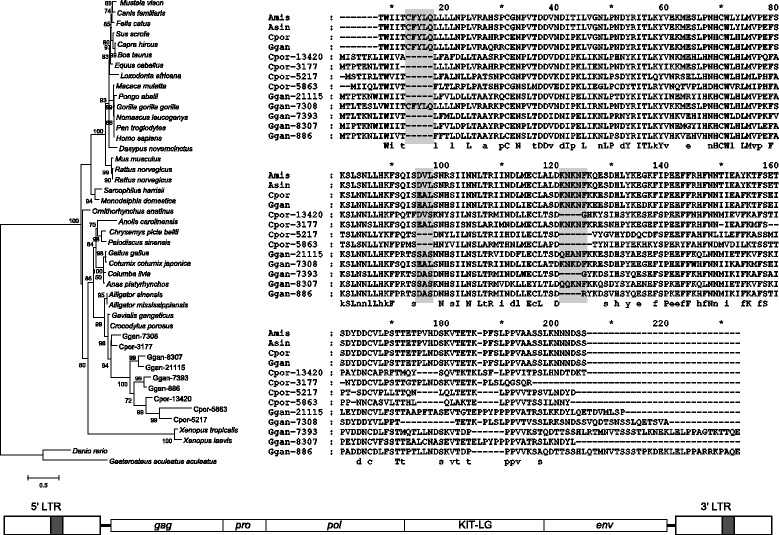
**Reconstructed KIT-ligand proteins from CrocERV29 and a simplified diagram of the provirus.** Maximum Likelihood phylogenies and sequence alignments were created from the reconstructed KIT-ligand proteins encoded by one lineage within CrocERV29. ERV IDs are provided within the tree and the alignment (see also Additional file 7). Four letter sequence names indicate the crocodilian species (‘Amis’ , *A. mississippiensis*; ‘Asin’ , *A. sinensis*; ‘Cpor’ , *C. porosus*; ‘Ggan’ , *G. gangeticus*). Shaded columns indicate the positions of indels within the alignment. Numbers within the tree indicate statistical support for the branches and the scale bars indicate branch length. Proviral structure is not to scale.

Some CrocERV29 copies contain an ORF between the *pol* and *env* genes that show similarity to the vertebrate KIT-ligand gene. This lineage within CrocERV29 is represented by the consensus sequence SFV1-21_Gav. We found nine copies retain an ORF corresponding to the entire KIT-ligand soluble form from the *C. porosus* and *G. gangeticus* genomes (Figure 3). These ORFs were surrounded by non-functional ERV sequence, with multiple nonsense mutations within the individual ERV ORFs. Despite this, we were able to reconstruct the retroviral domains from consensus sequences. Three short, in-frame indels were detected within at least eight of the KIT-ligand-like ORFs, although the impact of these on the function of potential proteins is unknown.

Pairwise genetic distances and phylogenetic analysis of the amino acid sequence of these ORFs compared with predicted KIT-ligand genes in the crocodilian genomes show that the *C. porosus* and *G. gangeticus* KIT-ligand genes are more closely related to each other than to the ERV copies. When compared with KIT-ligand transcripts from other species, all the crocodilian sequences, including the ERV sequences, formed a monophyletic sister clade to avian and reptilian KIT-ligand sequences (Figure 3). Codon based-Z tests suggest that purifying selection is the main selective force acting on these ORFs (*C. porosus*: *p* < 0.01, test statistic = 5.367; *G. gangeticus*: *p* < 0.01, test statistic = 4.561).

### Capture of nectin3 by CrocERV31

**Figure 4:**
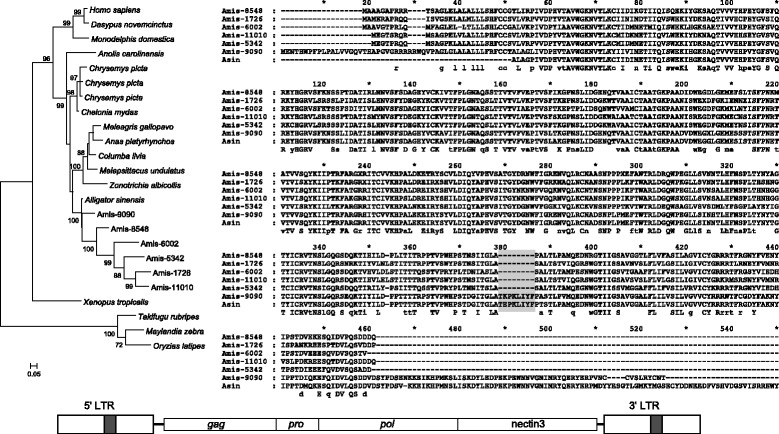
**Reconstructed nectin3 proteins from CrocERV31 and a simplified diagram of the provirus.** Maximum Likelihood phylogenies and sequence alignments were created from the reconstructed nectin3 proteins encoded by one lineage within CrocERV31. ERV IDs are provided within the tree and the alignment (see also Additional file 8). ‘Amis’ stands for *A. mississippiensis* and ‘Asin’ for *A. sinensis*. Shaded columns indicate the positions of indels within the alignment. Numbers within the tree indicate statistical support for the branches and the scale bars indicate branch length. Proviral structure is not to scale.

Another case of gene capture was observed in one lineage within CrocERV31, represented by six ERV insertions from *A. mississippiensis* (Figure 4), and the consensus sequences SFV1-5B_AMi and SFV1-5C_AMi. These sequences encode a protein derived from the nectin3 gene replacing the envelope protein. SFV1-5_AMi and SFV1-10_AMi lack an ORF for nectin3 although they are closely related to SFV1-5B_AMi and SFV1-5C_AMi. SFV1-10_AMi has an ORF for *env* and SFV1-5_AMi was revealed to be a deletion derivative of SFV1-5C_AMi.

The predicted protein contains three immunoglobulin-like domains, and spans the length of the reconstructed *A. sinensis* gene, suggesting that it also represents a captured host mRNA. The nectin3 gene of *A. mississippiensis* has not been sequenced completely, but the sequenced exons are >99% identical to those of the *A. sinensis* gene. The protein coded by SFV1-5C_AMi is ~76% identical to the nectin3 protein from *A. sinensis*. The ORF from the ERV insertion AKHW01077532 is more similar to the *Alligator* nectin3 protein than the proteins coded by the other CrocERV31 copies; the other five copies contain a deletion corresponding to the peptides EPKLIYFP. The genome of *A. sinensis* also contains an ERV4 group (SFV1-5_ASi) that encodes a nectin3-derived protein, indicating that the gene capture occurred before the speciation of *A. mississippiensis* and *A. sinensis* although none of SFV1-5_ASi copies retains a complete nectin3 ORF. This may be due to the low sequence coverage of the *A. sinensis* genome. Phylogenetic analyses of these sequences also supported a clustering of ERV derived sequences with the *A. sinensis* nectin3 protein (Figure 4). Codon based-Z tests suggest that these ORFs are subject to purifying selection (*p* < 0.01, test statistic = 7.529).

## Discussion

### Crocodilian ERVs may represent ancestral retroviral states

ERVs in crocodilians appear to be restricted to ERV1, ERV3, ERV4, and a small number of intermediate lineages. Unsurprisingly, ERV1 and ERV4 were the predominant lineages in the genomes, with 15 and 22 groups respectively. This is in agreement with previous studies that have identified a large number of ERV1 and ERV4 insertions across crocodilian species [2,4].

The crocodilian ERVs displayed very weak associations with ERVs from other taxa and exogenous retroviral genera, suggesting that ancient ERV insertions represent intermediates or novel lineages between the currently recognised taxa. Notably, the crocodilian ERVs tended to cluster separately from mammalian ERVs. The current findings are in accordance with previous studies where it was suggested that phylogenetic and evolutionary distance between potential host species may affect the potential distribution of ERV and retroviral linages [1,3], and suggest that the distinction between crocodilian and mammalian ERVs is the result of co-evolution between retroviruses and their host lineages.

Most of the ERV groups defined herein appear to have undergone very low levels of replication, with only a few groups represented by more than 50 insertions across the three genomes, even when less intact sequences were included. A small number of these groups appear to have undergone a greater degree of replication. The reasons behind this disparity is unclear, although differences in pathogenicity and virulence of the infecting exogenous retroviruses might be a contributing factor [22]. However, many groups also appear to be remnants of ancient retroviral infections, predating divergence of the major crocodilian lineages. Thus, it is possible that there are more degenerate insertions present that were not detected or included due to accumulation of mutations or loss of coding domains. Given observed correlations between transposable element activity and speciation events [29,30], it is also possible that this greater level of replication corresponds to significant periods of radiation and speciation in ancient crocodilians.

A large number of ERV groups were recovered from all three genomes although the degree of degradation observed in individual proviruses suggests that most of these are ancient infections. Surprisingly, a large number of ERV groups from ERV1 and ERV4 were found to be species specific, even when the search was expanded to include less intact ERVs. This implies that the exogenous retroviruses that gave rise to these endogenous groups were active relatively recently in crocodilian evolution. This is particularly significant for the ERV4 lineage as no exogenous counterpart has been described for these proviruses. That these insertions have maintained some capacity for replication suggests a low level of pathogenicity and virulence [22]. This in turn may also support the suggestion that these elements represent a less pathogenic precursor to modern retroviruses.

Crocodilian ERV3 groups were surprisingly diverse, with sequences clustering in two distinct groups. All three of these sequences, and a number of other ERV3 sequences within both groups encode a recognisable *env* (Figure 2), suggesting that the lack of *env* observed within a number of mammalian ERVs [12,16,31] is a derived characteristic of some lineages, and not necessarily a feature of the entire class. Likewise CrocERV 16 and 17, along with the avian ERV3 sequences, lack a dUTPase downstream of integrase. However, dUTPase is present in ERV3 sequences from a number of species, including mammals, crocodilians, turtles, and frogs suggesting that acquisition of this domain may predate the radiation of tetrapods. It is possible that this arose through horizontal transfer although the direction of this and the origin of the dUTPase containing lineage remains unclear.

### Differences in ERV complement between crocodilian species

Crocodilian genomes appear to contain a lower estimated percentage ERV content to that of most other characterised vertebrate species (Table 2). While the exact biological reasons behind this low ERV complement are not obviously apparent, genome biology and the genomic environment may play a role in determining the final ERV complement of a genome. Acquisition of specific control mechanisms, exaptation of ERV domains, and the insertion location can all dictate the preservation or removal of ERVs from a genome. It has also been suggested that some species, notably *Canis familiaris* (dog) and avians (represented here by *G. gallus*; chicken), may have additional mechanisms for purging ERVs from the genome or the restriction of retroviral activity [32]. As such, it is possible that similar mechanisms have evolved in crocodilians. Unfortunately, as with *C. familiaris*, the paucity of retroviral data in reptilians, and the current limited understanding of crocodilian genome biology limits the extent to which further conclusions can be drawn on this.

**Table 2:** **Estimated ERV content based on retroviral chains, and a comparison with previous estimates and other species**

**Species**	**Common name**	**% ERV chains in genome**	**% ERVs in genome**	**Reference**
*A. mississippiensis*	American alligator	0.25%	1.88%	[21]
*C. porosus*	Saltwater crocodile	0.26%	1.63%	[21]
*G. gangeticus*	Gharial	0.14%	1.22%	[21]
*Anolis carolinensis*	Green anole		3.00%	[33]
*Bos taurus*	European cattle	0.36%	4.29%	[34]
*Canis familiaris*	Dog	0.15%		[32]
*Danio rerio*	Zebrafish	0.80%		[32]
*Gallus gallus*	Chicken	0.20%	2.90%	[32,35]
*Homo sapiens*	Human	0.80%	8.29%	[22,36]
*Monodelphis domestica*	Opossum	2.00%	10.64%	[32,37]
*Mus musculus*	Mouse	2.00%	9.22%	[32,36]
*Xenopus tropicalis*	Western clawed frog		0.12%	[38]

Surprisingly, the estimated proportion of ERV chains in the genomes of the three crocodilian species appeared to vary greatly between species, with the predicted content of the *A. mississippiensis* and *C. porosus* genomes double that of the *G. gangeticus* genome. Interspecies variation in ERV content due to differing levels of ERV proliferation and loss as result of ERV evolution within host genomes is likely to be present, although it is likely to have a much lesser impact on the variation observed compared with genome contiguity and coverage [39,40]. *A. mississippiensis* was the most advanced of the three genomes, both in terms of contiguity as well as annotation (see also Table 1), and consequently, is more likely to be representative of the actual crocodilian genomes. The more fragmented nature of the *G. gangeticus* genome may reduce the ability of RetroTector to detect ERVs [22,32] due to potential fragmentation of the ERV chains, leading to lower estimates of ERV number and content.

Interestingly, we were able to recover lineages from the *Gammaretrovirus*-like ERV1 group, CrocERV5, from *A. mississippiensis*. This was unexpected as this lineage had previously been thought to be specific to *Crocodylidae* and *Gavialidae* [4]. The presence of three well preserved lineages within *A. mississippiensis* and *C. porosus*, and one less conserved lineage is present in *G. gangeticus* suggests either the presence of species or host family specific sublineages within this ERV group, or concurrent infection by closely related strains of the same exogenous retrovirus. While it is not possible to determine the most likely route of differentiation among the three genomes, both scenarios support the observed common ancestry of CrocERV5.

### Insights into the origin and evolution of ERVs

The retrieval of divergent ERVs from crocodilians demonstrates the diversity of ERVs present in non-mammalian vertebrates and highlights the importance of characterising ERVs from various vertebrate taxa for a better understanding of the origin and evolution of retroviruses. There has been some debate over the likely root of the retroviral evolutionary tree, largely spurred by the use of reverse transcriptase across retroviruses and ERVs, the *Gypsy* and *Ty1/copia* retroelements, and reverse transcribing DNA viruses such as the *Caulimoviridae* [41-43]. The long standing, and commonly accepted theory is the evolution of retroviruses from *Gypsy*-type retrotransposons following acquisition of *env*, facilitating extracellular movement and production of infectious particles [22,42,44], although the lineage or lineages of *Gypsy* that contributed to the birth of retroviruses is still debatable. A second hypothesis has recently been proposed, stating that three classes of retroviruses (Classes I, II and III) were derived from three different *Gypsy* retrotransposons and acquired their *env* proteins independently [24].

Our findings support the traditional theory that the currently recognised exogenous retroviral genera have evolved through a process of gradual evolution from a single retroviral precursor [1,9]. Our data suggests that Class I and Class II retroviruses are more derived retroviral groups and non-Class I/Class II retroviruses represent the ancient retroviral diversity. Our phylogeny supports monophyly of Class I retroviruses and of Class II retroviruses, but not of Class III (ERV3 and *Spumaviruses*) and other non-Class I/Class II retroviruses, indicating that our ERV4 sequences are not a divergent lineage of Class III retroviruses. Shared proviral characteristics such as the lack of zinc-finger motifs in the *gag* protein and the absence of GPY/F-like motif downstream of integrase in ERV4 may support the common ancestry of ERV3, ERV4 and *Spumaviruses*, but further analysis is necessary to clarify their relationships. ERVs related to Ovex1 and SnRV also remain to be classified. Some of these encode a GPY/F-like motif downstream of integrase and/or a zinc-finger motif in the *gag* protein. Finally, the presence of *env* proteins coded by most of non-Class I/Class II retroviruses supports the acquisition of *env* protein by the common ancestor of all retroviruses, lending support to the traditional hypothesis of a shared common ancestor of all retroviral and ERV classes.

### Two crocodilian ERV groups have captured host mRNAs

The acquisition of an additional ORF through capture of host mRNA is a relatively uncommon occurrence. This usually results in the deletion of part of the internal viral coding domains, rendering the resulting provirus incapable of autonomous replication [19]. The KIT-ligand containing lineage within CrocERV29 is highly unusual in this respect, as it appears that incorporation of the KIT-ligand mRNA has taken place between the *pol* and *env* genes without significant loss of viral coding regions. To date, the only other documented occurrences are in the replication competent Rous sarcoma virus (RSV) and the piscine retrovirus, Walleye epidermal hyperplasia virus (WEHV). RSV encodes an additional protein *Src*, a tyrosine kinase that stimulates uncontrolled mitosis of host cells [45,46]. WEHV encodes three additional ORFs, two of which, *orfA* and *orfB* encode cyclin D homologues [20,47]. Both of these genes play a role in cell division, and likely have similar action when expressed by infecting retroviruses, thereby providing abundant cells for fresh infection.

KIT-ligand, also known as stem cell factor (SCF), *steel* factor (SLF), or mast cell growth factor (MCGF), is a cytokine that binds to a tyrosine kinase receptor c-Kit, also called CD117 [48-50]. The KIT-ligands play an important role in a variety of functions ranging from gametogenesis, melanogenesis and haematopoesis [51]. Like *Src*, KIT-ligand and c-Kit show an association with cancer. The KIT-ligand gene locus was identified as a cancer susceptibility locus for human testicular germ cell tumors [52,53]. Similarly, a copy number variant near the KIT-ligand gene likely confers risk for canine squamous cell carcinoma of the digit [54]. c-Kit, is a proto-oncogene, meaning that overexpression or mutations of this protein can lead to cancer. Its viral homolog, v-Kit, was recovered from a recombinant oncogenic retrovirus Hardy-Zuckerman 4 feline sarcoma virus (Hz4-FeSV) [55].

The current findings provide the basis for further studies to investigate whether the KIT-ligands coded by CrocERV29 retain oncogenic properties. Also worth examining are the functionality and locations where the KIT-ligand retrocopies are expressed. It is possible that these retrocopies have been subfunctionalised with the original KIT-ligand gene, in a similar fashion to the two paralogous KIT-ligands in *D. rerio*, where these genes share complementary functions and display tissue specific expression patterns [56]. The length and completeness of the recovered ORFs suggest that at least some functionality has been retained, particularly given that the surrounding ERV sequences are no longer functional [22]. Further to this, the close relationships and clustering of these retrocopies suggests that this is an ancestral event, with the incorporation occurring prior to the emergence of the crocodile and gavial lineages. Thus, the conserved nature of these ORFs, combined with purifying selection suggests that these retrocopies may encode functional proteins. However, in the absence of available transcriptome data for these species, it is unclear whether these KIT-ligands represent an exapted retroviral gene capture or exploitation of host genes to facilitate retroviral replication.

The nectin3 containing lineages within CrocERV31 represent a more typical acquisition, whereby the host mRNA is incorporated at the expense of the *env* gene. Thus, these lineages are likely to have replicated by retrotransposition within the host genome. The nectin proteins form a family of integral molecules that belong to the immunoglobulin superfamily [57]. Nectin3 binds to nectin1 which acts as a poliovirus or alpha-herpesvirus receptor [58]. We can speculate that the captured nectin3 protein also binds to nectin1 and has contributed to the infection and proliferation of this ERV lineage. Similar to the KIT-ligand retrocopies, the intact nature of the nectin3 ORFs described herein, as well as evidence of purifying selection suggests that these have retained some functionality, and may warrant further investigation.

## Conclusions

Our study indicates that crocodilian ERVs stem from infection events by retroviruses from a wide variety of lineages, although the overall proportion of the crocodilian genomes that can be attributed to these elements does not differ greatly from other characterised species. There is evidence that a small number of crocodilian ERV groups have undergone significant levels of replication within crocodilian genomes at some stage in their evolution. In particular, the capture of host mRNA by two ERV lineages followed by the subsequent replication of these lineages merits further investigation, and highlights the potential impacts and significance of ERV replication and maintenance in crocodilians.

Using the resources generated here, it will be possible to extend ERV studies in crocodilians to assess the interactions of these ERVs with the crocodilian genomes, and the roles they may play in the biology of these species. Further investigation into the demographics of these ERVs may provide insights into the population demographics of ancient crocodilians and corroborate molecular and fossil evidence of crocodilian radiation. This study also provides a framework to facilitate further studies into crocodilian ERV diversification as well as other basal vertebrate species. Distributions of the ERV groups across the sequenced crocodilian taxa suggest that most of these are ancient integration events predating the divergence of the crocodilian families. The recovery of apparent intermediates between the major ERV classes highlights the need for detailed studies into the ERVs of the basal vertebrate families. Additionally, these data offer valuable insights into the proviral structure of ancient ERVs, and the possible mechanisms by which these elements have evolved from genomic retroelements to extracellular pathogens.

## Methods

### Recovery of ERV sequence data

Assembled scaffolds from the three crocodilian genomes were mined for ERV sequences using RetroTector and a chain cut-off of 250 to enable the detection of divergent proviruses [59]. Briefly, RetroTector identifies potential domains by similarity to known functional and structural motifs, and attempts to re-create the coding domains and identify the outer bounds of individual ERV sequences. Custom python scripts were used to retrieve and collate the RetroTector sequence data from each of the sequences of interest. Where duplicate sequences arose from the splitting of scaffolds by RetroTector, the predictions of internal domains were manually checked and the information from each entry was merged. The estimated proportion of each genome that was likely to be ERV related was calculated from the lengths of the ERV chains detected using RetroTector and the total length of the assembled scaffolds (Table 1). Individual sequences from each genome were identified by a four letter species designation based on the first two letters of the genus and species names followed by the scaffold number and ERV ID as classified by RetroTector. Under this system, “Almi” stands for *A. mississippiensis*, “Crpo” for *C. porosus*, and “Gaga” for *G. gangeticus*. We used the term “CrocERV” to define each of the ERV groups identified from this dataset.

Systematic screening of repetitive sequences was performed in parallel using custom-made scripts based on the methods described before [60]. The consensus sequences were derived using the majority rule applied to the corresponding sets of aligned copies, followed by manual inspection to remove frameshift and nonsense mutations introduced during consensus building. ERV sequences were extracted from the complete repeat dataset based on the sequence similarity to known ERV sequences in Repbase [61]. Some consensus sequences were reconstructed based on copies detected by RetroTector. Each sequence was classified based on the sequence similarity to known ERV sequences from non-crocodilians and classified crocodilian ERV sequences. These classifications are represented within the sequence names where ERVX indicates overall similarity to the respective ERV classes, and SFV (simian foamy virus-like) has been used to identify the ERV4 consensus sequences. Three-letter suffixes AMi/Ami, Crp, Gav show the origin of sequences which the consensus sequences were built from, *A. mississippiensis*, *C. porosus*, and *G. gangeticus* respectively, although the corresponding lineage is not necessarily distributed only in the single species. The suffix ‘Croc’ is used for the consensus sequences that were reconstructed from genomic sequences of multiple species. A different suffix system to that used for the RetroTector analysis was implemented to maintain consistency with other Repbase entries.

### Definition of ERV groups

ERV groups were defined from the RetroTector data based on the predicted amino acid sequences of *pol*. Due to the large number of insertions from all three genomes, only the sequences deemed to be ‘complete’ ERVs were used. These sequences were those where both LTRs and all four retroviral genes (*gag*, *pro*, *pol*, and *env*) could be predicted by RetroTector, and the retroviral domains reconstructed from the corresponding Repbase consensus sequences. Sequences with more than five consecutive ambiguous amino acid residues within *pol* were also excluded to ensure that fragmented insertions and potential assembly artefacts were not incorporated into the final dataset. While these criteria may bias analyses to insertions that are better preserved or more recently integrated, it also reduces the amount of sequence divergence and evolutionary ‘noise’ that may be introduced by the inclusion of highly degraded sequences. BLASTX [62] was used to classify the predicted *pol* sequences into the major ERV classes based on similarity to *pro-pol* and *pol* fragments recovered from previous studies [1-5]; Chong et al., unpublished data]. Sequences that showed no similarity to known crocodilian ERV fragments where then compared to other published sequences in GenBank and Repbase using the NCBI BLAST suite [63] and Censor [64]. Orthologous ERVs were identified using based on 80% sequence similarity across 500 bp of unambiguous, non-repetitive genomic sequence from either side of the identified insertion sites.

As commonly used to determine preliminary ERV lineages, phylogenetic trees were then created using Neighbour Joining, uncorrected sequence distances, and 1000 bootstrap replicates [5,9,32,34,35,65]. Nucleotide sequences within each of the major classes were aligned in MAFFT [66] using the E-INS-i algorithm, then trees were created using CLUSTALW [67]. Sequences from clades with more than 70% bootstrap support were then realigned and refined based on sequence similarity and conservation within *pol* such that sequences for each lineage were more similar to each other than those of other lineages. Amino acid sequence similarities within ERV groups and ERV classes were calculated using p-distances in MEGA5 [68].

We then used Censor to refine these groups based on similarity to the consensus sequences defined using the Repbase pipeline, such that each ERV group formed a monophyletic clade and were represented by at least one Repbase consensus sequence where the RT could be reconstructed without frameshift or nonsense mutations. The distribution of each ERV group was predicted based on the species that the ERV sequences were recovered from and confirmed by the Censor search with ERV consensus sequences as queries.

We also created a phylogeny of the consensus sequences compared to ERVs from other species, to assess the evolutionary relationships between these. For this, we extracted amino acid sequences for the full-length RT domains of non-crocodilian ERV lineages from Repbase. The final dataset comprised 420 sequences, including 35 sequences from exogenous retroviruses, 80 consensus sequences from the CrocERV groups defined above, 1 sequence of the Ovex1 gene from *G. gallus,* 2 ERV4 lineages from turtles, and 9 published avian ERV consensus sequences [69]. Sequences from the 22 *Gypsy* lineages defined by Llorens et al. [24,70] were included as an outgroup. Amino acid sequences were aligned by MAFFT using the E-INS-i algorithm. A Maximum Likelihood tree was constructed using the rtREV substitution matrix [71] in PhyML [72] and aLRT statistics [73] to indicate branch support (Figure 1; full phylogeny is included as Additional file 6: Figure S2). A simplified phylogeny was also created to determine the relationships between ERV3 lineages encoding *env* and dUTPase using subsets of these sequences and the same methods as described above (Figure 2), and comprised 38 ERV3 and spumaviral sequences along with 3 *Gypsy* elements.

We estimated insertion dates of these ERV groups using the average sequence differences between LTRs of proviral LTRs predicted by RetroTector. LTR sequences from each insertion were aligned using CLUSTALW. After removal of sequence pairs that could not be aligned due to indels, genetic distances were calculated using the Kimura two parameter model [74] in MEGA5 and averaged for each ERV group. Ages were calculated using *T = d/2r* where *T* is the estimated insertion time, *d* is the genetic distance between the 5` and 3` LTRs, and *r* is the rate of nucleotide substitutions per site per year (s/s/y). A neutral substitution rate of 3.9 × 10^−10^ [21] (based on a mutation rate of 7.9 × 10^−9^ and a generation interval of 20 years) was used for the calculations, although this is a rough estimate due to the difficulty estimating generation intervals for crocodilians.

### Characterisation of KIT-ligand genes from crocodilians

The KIT-ligand gene sequences predicted by the Crocodilian Genome Sequencing Consortium were corrected based on the comparison with KIT-ligand proteins from other vertebrate species (Additional file 1: Table S3, Additional file 7). The predicted protein sequences were aligned with representative KIT-ligand proteins and amino acid sequences encoded by CrocERV29 using MAFFT and the L-INS-i algorithm. Reconstructed amino acid sequences were then realigned with vertebrate KIT-ligand proteins using MUSCLE [75] and a Maximum Likelihood phylogeny was constructed using PhyML and the JTT + G model as determined by ModelGenerator. The ORFs from each species were assessed for evidence of selection using the Codon-Z test implemented in MEGA5.

### Characterisation of the nectin3 gene from *Alligator sinensis*

While retrieving the host copies of the captured ORFs, it was observed that the nectin3 gene from *A. mississippiensis* has not been completely sequenced. In order to recover a crocodilian copy of the gene for comparison, we used the recently published genome of *A. sinensis* [76]. TBLASTN was used to locate the homologous sequence using the protein sequence of nectin3 from *Melopsittacus undulatus* (Budgerigar, NCBI: XP_005144774) as a query. We extracted the corresponding genomic region and plotted the *M. undulatus* protein sequence onto the *A. sinensis* genomic sequence with the aid of prosplign (http://www.ncbi.nlm.nih.gov/sutils/static/prosplign/prosplign.html) to characterise the exon-intron structure (Additional file 8). The predicted protein sequence was aligned with representative nectin3 proteins and proteins encoded by CrocERV31 copies using MAFFT and the L-INS-i algorithm. Phylogenetic analysis was carried as for the KIT-ligand sequences using the JTT + G model of amino acid substitution (Additional file 1: Table S4). ORFs were tested for evidence of selection as described above for the KIT-ligand sequences.

## Additional files

Additional file 1: Table S1. Summary of ERV insertions with a chain score >300 detected from each of the genomes, and an approximation of the ERV content. Supplementary **Table S2**: Summary of ERV families and their predicted distribution among crocodilians. Supplementary **Table S3**: UniProt ID, and scientific and common names of additional species used for phylogenetic analysis of the KIT-ligand-like ORF. Supplementary **Table S4**: UniProt ID, and scientific and common names of additional species used for phylogenetic analysis of the Nectin3-like ORF. Additional file 2:
**Repbase_entries.xlsx.** Summary of the consensus sequences submitted to Repbase including internal and LTR sequences. Entries are available from http://www.girinst.org/repbase/index.html. Additional file 3: Figure S1. Clustering of sequences within ERV groups as defined using complete ERV sequences. Additional file 4:
**RetroTector_ERV_sequences.csv.** Overview of individual sequence identifiers and genomic locations of each intact insertion used in the analyses. Also includes the corresponding CrocERV family, ERV class, and predicted PBS, PPT and TSD sequences. Additional file 5:
**CrocERV_Pol_proteins.fas.** Fasta file containing the 295 *pol* protein sequences used to define the CrocERV families. Additional file 6: Figure S2. Classification and likely relationships between the ERV families and ERVs from other species (complete phylogeny with sequence IDs). Additional file 7:
**KIT-ligand.pdf.** KIT-ligand genes from four species of crocodilians and KIT-ligand-like sequences encoded by copies of CrocERV29. This file contains: Scaffold number, exon-intron locations, cDNA sequences, and amino acid sequences of the KIT-ligand genes from *A. mississippiensis*, *A. sinensis*, *C. porosus*, and *G. gangeticus*. Reconstructed cDNA and amino acid sequences of the KIT-ligand-like retrocopies from CrocERV29 insertions. Additional file 8:
**Nectin3.pdf.** Nectin3 genes from *A. sinensis* and nectin3-like sequences encoded by copies of CrocERV31. This file contains: Scaffold number, exon-intron locations, cDNA sequence, and amino acid sequence of the nectin3 gene from *A. sinensis*. Reconstructed cDNA and amino acid sequences of the nectin3-like retrocopies from CrocERV31 insertions.
