# Supplementary material for: Evolution and gene capture in ancient endogenous retroviruses - insights from the crocodilian genomes
Source: Retrovirology. 2014 Dec 12;11:71. doi: 10.1186/s12977-014-0071-2 (PMC4299795; doi:10.1186/s12977-014-0071-2)
Supplement: Additional file 1: Table S1. — Summary of ERV insertions with a chain score >300 detected from each of the genomes, and an approximation of the ERV content. Supplementary Table S2: Summary of ERV families and their predicted distribution among crocodilians. Supplementary Table S3: UniProt ID, and scientific and common names of additional species used for phylogenetic analysis of the KIT-ligand-like ORF. Supplementary Table S4: UniProt ID, and scientific and common names of additional species used for phylogenetic analysis of the Nectin3-like ORF. [file 12977_2014_71_MOESM1_ESM.pdf]

Additional File 1: Supplementary Tables

This file contains:

- **Supplementary Table S1:** Summary of ERV insertions with a chain score >300 detected from each of the genomes, and an approximation of the ERV content.
- **Supplementary Table S2:** Summary of ERV families and their predicted distribution among crocodilians.
- **Supplementary Table S3:** UniProt ID, and scientific and common names of additional species used for phylogenetic analysis of the KIT-ligand-like ORF.
- **Supplementary Table S4:** UniProt ID, and scientific and common names of additional species used for phylogenetic analysis of the Nectin3-like ORF.

Supplementary Table S1: Summary of ERV insertions with a chain score >300 detected from each of the genomes, and an approximation of the ERV content.

	A. mississippiensis	*C. porosus*	*G. gangeticus*
Genome size[a]	2.5	2.78	2.5
Version[b]	aMiss_0.2	croc_sub2	ggan_v0.2
Length of assembly	2,144,153,452	2,123,474,087	2,882,656,219
Number of scaffolds	8,897	23,365	47,351
Estimated proportion of genome in assembly	0.86×	0.76×	1.15×
Number of ERV chains (>300)	722	772	561
Complete ERVs	154	211	106
Both LTRs	492	465	293
One LTR	80	77	66
No LTRs	150	230	202
Solo LTRs	79,445	119,753	140,412
Estimated ERV content[c] (ERV chains)	1.88% (0.25%)	1.63% (0.26%)	1.22% (0.14%)

[a] Estimates of genome size taken from St John et al. [1].
[b] Genomes were downloaded before the 'freeze' of assembly and refinement. Therefore these may not represent the latest, or the published version of the genomes.
[c] Estimates and breakdown of all transposable elements available in Green et al. [2].

Supplementary Table S2: Summary of ERV families and their predicted distribution among crocodilians. The distribution Crocodylia refers to families found in all three species whereas *Longirostres* refers to those only found in both *C. porosus* and *G. gangeticus*. *Alligatoridae*, *Crocodylidae*, and *Gavialidae* refer to the predicted distributions of families found only in a single species.

Family	ERV Class	Distribution	No. seqs	TSD length (bp)	Estimated Age (MY)	Pol similarity	Corresponding RepBase consensus	Additional characteristics
CrocERV1	ERV1	Longirostres	67	4	80.00	0.63	ERV1-6_Crp<br>ERV1-1_Gav	
CrocERV2	ERV1	Alligatoridae	5	4	39.74	0.83	ERV1-6_AMi	
CrocERV3	ERV1	Alligatoridae	6	4	39.10	0.64	ERV1-3_Ami<br>ERV1-3B_AMi	
CrocERV4	ERV1	Longirostres	3	4	67.31	0.68	ERV1-2_Gav	
CrocERV5	ERV1	Crocodylia	7	4	28.21	0.72	ERV1-1_Ami<br>ERV1-2_Crp<br>ERV1-7_Crp	
CrocERV6	ERV1	Longirostres	4	4		0.66	ERV1-5_Crp<br>ERV1-5B_Crp	
CrocERV7	ERV1	Longirostres	6	4	37.82	0.74	ERV1-1_Crp	
CrocERV8	ERV1	Longirostres	8	4	108.21	0.62	ERV1-3_Crp	
CrocERV9	ERV1	Alligatoridae	4	4	37.61	0.77	ERV1-4_AMi	
CrocERV10	ERV1	Alligatoridae	2	4	56.41	0.80	ERV1-9_AMi	
CrocERV11	ERV1	Crocodylia	9	4	64.10	0.65	ERV1-7_Ami<br>ERV1-12_AMi	
CrocERV12	ERV1	Crocodylia	33	4	70.15	0.61	ERV1-2_Ami<br>ERV1-4_Crp<br>ERV1-4_Gav	
CrocERV13	ERV1	Alligatoridae	1	4			ERV1-10_Ami	
CrocERV14	ERV1	Alligatoridae	1	4			ERV1-8_AMi	
CrocERV15	ERV1	Crocodylidae	1	4			ERV1-8_Crp	
CrocERV16	ERV3	Crocodylia	4	6	62.82	0.50	ERV3-2_Crp	Lacks dUTPase<br>Unusual TSD length
CrocERV17	ERV3	Crocodylia	6	5		0.51	ERV3-2_AMi	Lacks dUTPase
CrocERV18	ERV4	Longirostres	11	5	85.26	0.66	SFV1-2_Crp<br>SFV1-15_Gav	
CrocERV19	ERV4	Longirostres	9	5	221.15	0.59	SFV1-3_Crp<br>SFV1-15_Crp<br>SFV1-5_Gav<br>SFV1-18_Gav	
CrocERV20	ERV4	Alligatoridae	7	5	39.23	0.49	SFV1-1_Ami<br>SFV1-8_Ami<br>SFV1-9_Ami<br>SFV1-11_Ami<br>SFV1-16_Ami	

						SFV1-20_AMi	
CrocERV21	ERV4	Crocodylidae	3	5	64.10	0.65 SFV1-12_Crp	
CrocERV22	ERV4	Longirostres	8	5	77.56	0.52 SFV1-22_Gav	
CrocERV23	ERV4	Longirostres	2	5	67.95	0.65 SFV1-19_Crp	
CrocERV24	ERV4	Crocodylidae	3	5	57.69	0.75 SFV1-10_Crp	
CrocERV25	ERV4	Longirostres	4	5		0.65 SFV1-1_Crp	
						SFV1-2_Gav	
CrocERV26	ERV4	Longirostres	8	5		0.55 SFV1-7_Crp	
						SFV1-8_Crp	
CrocERV27	ERV4	Longirostres	4	5	0.00	0.39 SFV1-20_Crp	
CrocERV28	ERV4	Gavialidae	3	5	107.69	0.70 SFV1-28_Gav	
CrocERV29	ERV4	Longirostres	31	5	61.06	0.47 SFV1-4_Crp	One lineage encodes an additional ORF showing similarity to KIT-ligand
						SFV1-5_Crp	
						SFV1-6_Crp	
						SFV1-11_Crp	
						SFV1-14_Crp	
						SFV1-14B_Crp	
						SFV1-17_Crp	
						SFV1-1_Gav	
						SFV1-4_Gav	
						SFV1-8_Gav	
						SFV1-9_Gav	
						SFV1-10_Gav	
						SFV1-12_Gav	
						SFV1-13_Gav	
						SFV1-14_Gav	
						SFV1-16_Gav	
						SFV1-17_Gav	
						SFV1-19_Gav	
						SFV1-20_Gav	
						SFV1-21_Gav	
						SFV1-23_Gav	
						SFV1-24_Gav	
						SFV1-25_Gav	
						SFV1-25B_Gav	
CrocERV30	ERV4	Alligatoridae	8	5	45.94	0.66 SFV1-4_Ami	
						SFV1-7_AMi	
CrocERV31	ERV4	Alligatoridae	6	5	63.68	0.72 SFV1-5_Ami	One lineage encodes nectin3-like ORF in place of *env*
						SFV1-10_AMi	
CrocERV32	ERV4	Alligatoridae	3	5	30.13	0.72 SFV1-3_Ami	
						SFV1-17_AMi	
CrocERV33	ERV4	Alligatoridae	6	5	91.67	0.63 SFV1-2_Ami	

							SFV1-6_AMi	
CrocERV34	ERV4	Longirostres	4	5	47.44	0.53	SFV1-11_Gav	
							SFV1-11B_Gav	
CrocERV35	ERV4	Crocodylidae	1	5	61.54		SFV1-18_Crp	
CrocERV36	ERV4	Crocodylia	1	5	60.26		SFV1-18_AMi	
							SFV1-21_Crp	
CrocERV37	Unknown	Crocodylia	2	4		0.68	ERV-3_AMi	
							ERV-2B_Crp	
CrocERV38	Unknown	Crocodylia	2	5		0.85	ERV-5_Ami	
CrocERV39	Unknown	Crocodylia	1	4			ERV-1_Crp	
							ERV-2_AMi	
CrocERV40	Unknown	Crocodylia	1	5			ERV-1_AMi	
							ERV-7_Crp	
CrocERV41	ERV3	Crocodylia	N/A	5			ERV3-1_AMi	Encodes dUTPase
CrocERV42	ERV4	Longirostres	N/A	5			SFV1-16_Crp	
							SFV1-27_Gav	
CrocERV43	ERV4	Longirostres	N/A	5			SFV1-3_Gav	
CrocERV44	ERV4	Gavialidae	N/A	5			SFV1-6_Gav	
CrocERV45	Unknown	Crocodylia	N/A	5			ERV-4_AMi	

Supplementary Table S3: UniProt ID, and scientific and common names of additional species used for phylogenetic analysis of the KIT-ligand-like ORF.

UniProt/GenBank Accession number	Scientific name	Common name
A8WC00	*Gasterosteus aculeatus aculeatus*	Three-spined stickleback
ADT62517	*Anas platyrhynchos*	Mallard duck
B4F6K4	*Xenopus tropicalis*	Western clawed frog
F6SDB0	*Macaca mulatta*	Rhesus macaque
F7C5I3	*Ornithorhynchus anatinus*	Platypus
F7EP53	*Monodelphis domestica*	Opossum
G1R0E6	*Nomascus leucogenys*	White-cheeked gibbon
G3S4K2	*Gorilla gorilla gorilla*	Gorilla
G3VV70	*Sarcophilus harrisii*	Tasmanian devil
H2NI76	*Pongo abelii*	Sumatran orangutan
H2Q6K8	*Pan troglodytes*	Chimpanzee
K7FR23	*Pelodiscus sinensis*	Chinese softshell turtle
NP_001018133	*Danio rerio*	Zebrafish
P20826	*Mus musculus*	House mouse
P21581	*Rattus norvegicus*	Brown rat
P21583	*Homo sapiens*	Human
P79169	*Felis catus*	Domestic cat
Q06220	*Canis familiaris*	Domestic dog
Q09108	*Gallus gallus*	Chicken
Q28132	*Bos taurus*	Cow
Q29030	*Sus scrofa*	Pig
Q7ZXV0	*Xenopus laevis*	African clawed frog
Q90314	*Coturnix coturnix japonica*	Japanese quail
Q95M19	*Capra hircus*	Goat
Q95MD2	*Equus caballus*	Horse
Q95N18	*Mustela vison*	American mink
XP_003405659	*Loxodonta africana*	African elephant
XP_004459893	*Dasypus novemcinctus*	Nine-banded armadillo
XP_005279181	*Chrysemys picta bellii*	Painted turtle
XP_005508365	*Columba livia*	Rock dove

Supplementary Table S4: UniProt ID, and scientific and common names of additional species used for phylogenetic analysis of the Nectin3-like ORF.

UniProt/GenBank Accession number	Scientific name	Common name
EMP37855	*Chelonia mydas*	Green turtle
NP_056295	*Homo sapiens*	Human
XP_001362370	*Monodelphis domestica*	Opossum
XP_002940155	*Xenopus tropicalis*	Western clawed frog
XP_003202854	*Meleagris gallopavo*	Wild turkey
XP_003219129	*Anolis carolinensis*	Carolina anole
XP_003968614	*Takifugu rubripes*	Pufferfish
XP_004075606	*Oryzias latipes*	Japanese rice fish
XP_004456966	*Dasypus novemcinctus*	Nine-banded armadillo
XP_004558293	*Maylandia zebra*	Zebra mbuna
XP_005024167	*Anas platyrhynchos*	Mallard duck
XP_005144774	*Melepsittacus undulatus*	Budgerigar
XP_005283666	*Chrysemys picta*	Painted turtle
XP_005492614	*Zonotrichia albicollis*	White-throated sparrow
XP_005502646	*Columba livia*	Rock dove

1. St John J, Braun E, Isberg S, Miles L, Chong A, Gongora J, Dalzell P, Moran C, Bed'Hom B, Abzhanov A, et al: **Sequencing three crocodilian genomes to illuminate the evolution of archosaurs and amniotes.** *Genome Biol* 2012, **13:**415.
2. Green RE, Braun EL, Armstrong J, Earl D, Nguyen N, Hickey G, Vandewege MW, St John JA, Capella-Gutiérrez S, Castoe TA, et al: **The genomes of three crocodilians provide insight into archosaur evolution.** *Science* Submitted.
