# Supplementary material for: Evolution and gene capture in ancient endogenous retroviruses - insights from the crocodilian genomes
Source: Retrovirology. 2014 Dec 12;11:71. doi: 10.1186/s12977-014-0071-2 (PMC4299795; doi:10.1186/s12977-014-0071-2)
Supplement: Additional file 3: Figure S1. — Clustering of sequences within ERV groups as defined using complete ERV sequences. [file 12977_2014_71_MOESM3_ESM.pdf]

**Additional File 3:** Supplementary Figure S1

This file contains:

- **Supplementary Figure S1:** Clustering of sequences within ERV groups as defined using complete ERV sequences.

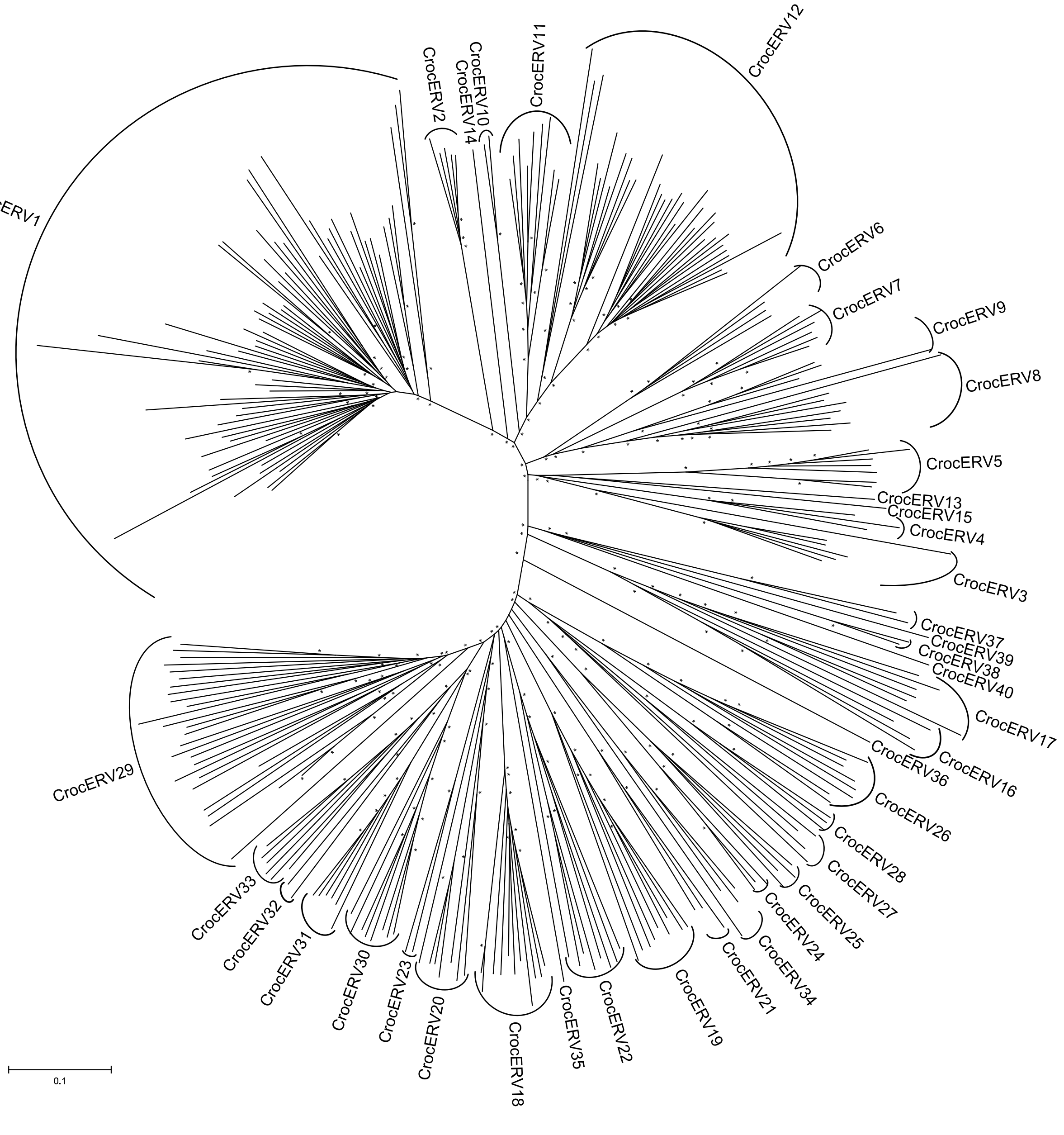


**Supplementary Figure S1:** Clustering of sequences within the ERV groups as defined using complete ERV sequences. An unrooted Neighbour Joining tree was created from amino acid alignments of *pol*. Sequence IDs have been removed for clarity and only the group names are shown. The scale bar on the left indicates branch lengths and asterisks within the tree indicate bootstrap support values greater than 70%.
