# Supplementary material for: Evolution and gene capture in ancient endogenous retroviruses - insights from the crocodilian genomes
Source: Retrovirology. 2014 Dec 12;11:71. doi: 10.1186/s12977-014-0071-2 (PMC4299795; doi:10.1186/s12977-014-0071-2)
Supplement: Additional file 6: Figure S2. — Classification and likely relationships between the ERV families and ERVs from other species (complete phylogeny with sequence IDs). [file 12977_2014_71_MOESM6_ESM.pdf]

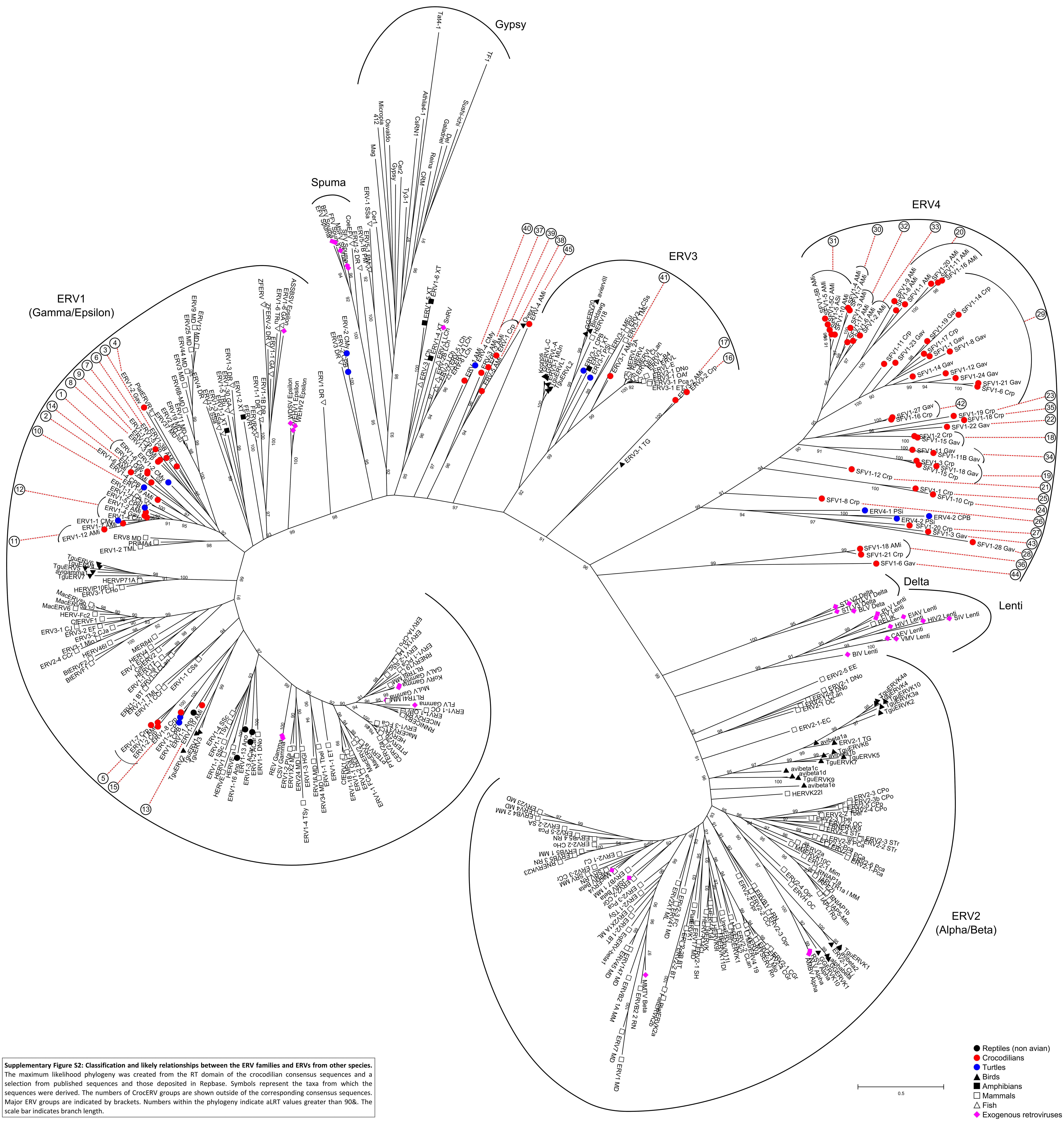

Supplementary Figure S2: Classification and likely relationships between the ERV families and ERVs from other species. The maximum likelihood phylogeny was created from the RT domain of the crocodilian consensus sequences and a selection from published sequences and those deposited in Repbase. Symbols represent the taxa from which the sequences were derived. The numbers of CrocERV groups are shown outside of the corresponding consensus sequences. Major ERV groups are indicated by brackets. Numbers within the phylogeny indicate aLRT values greater than 90&. The scale bar indicates branch length.
