# Supplementary material for: Evolution and gene capture in ancient endogenous retroviruses - insights from the crocodilian genomes
Source: Retrovirology. 2014 Dec 12;11:71. doi: 10.1186/s12977-014-0071-2 (PMC4299795; doi:10.1186/s12977-014-0071-2)
Supplement: Additional file 7: — KIT-ligand.pdf. KIT-ligand genes from four species of crocodilians and KIT-ligand-like sequences encoded by copies of CrocERV29. This file contains: Scaffold number, exon-intron locations, cDNA sequences, and amino acid sequences of the KIT-ligand genes from A. mississippiensis, A. sinensis, C. porosus, and G. gangeticus. Reconstructed cDNA and amino acid sequences of the KIT-ligand-like retrocopies from CrocERV29 insertions. [file 12977_2014_71_MOESM7_ESM.pdf]

Additional File 7: KIT-ligand genes from four species of crocodilians and KIT-ligand-like sequences encoded by copies of CrocERV29.

This file contains:

- Scaffold number, exon-intron locations, cDNA sequences, and amino acid sequences of the KIT-ligand genes from *A. mississippiensis*, *A. sinensis*, *C. porosus*, and *G. gangeticus*.
- Reconstructed cDNA and amino acid sequences of the KIT-ligand-like retrocopies from CrocERV29 insertions.

Crocodilian KIT-ligand genes

Alligator mississippiensis: scaffold 3372 (GenBank: JH733102)

Exon-intron structure:

Exon1	44030	44143
Exon2	60206	60268
Exon3	67304	67474
Exon4	72815	72974
Exon5	74407	74486

```
>Alligator_mississippiensis_cDNA
ACTTGGATTATCACTTGCTTTTATCTTCAGCTGCTCCTACTTAATCCACTTGTCAGAGCTCATAGTCCCTGTGGG
AATCCAGTGACAGATGATGTGAATGACATTACAATATTGGTTGGAAATCTTCCAAATGATTATAGGATAACCCTT
AAATATGTCGAGAAGATGGAAAGTCTGCCTAATCACTGTTGGTTGTATTTGATGGTGCCTGAATTTTCAAAGAGT
CTGAGTAATCTGCTCCACAAATTTTCACAAATATCTGATGTTTTAAGCAACCGTTCAATCATCAATAATCTCACA
AGGATAATTAATGATCTTATGGAATGTCTAGCATTAGATAAAAATAAGAATTTTAAACAAGAAAGCGATCACCTG
TATAAAGAAGGCAAGTTCATTCCTGAGGAATTCTTTAGGCATTTTAACAACACCATTGAAGCCTATAAGACGTTT
TCAGAAACATCAGATTATGATGACTGTGTTCTGCCTTCAACAACAGAAACACCAGTTCATGATTCCAAGGTCACT
GAAACAAAGCCATTTTCATTACCTCCTGTTGCTGCCAGTTCCCTTAAGAATAACAATGATAGCAGT

>Alligator_mississippiensis_Protein
TWIITCFYLQLLLLNPLVRAHSPCGNPVTDDVNDITILVGNLPNDYRITLKYVEKMESLPNHCWLYLMVPEFSKS
LSNLLHKFSQISDVLSNRSIINNLTRIINDLMECLALDKNKNFKQESDHLYKEGKFIPEEFFRHFNNTIEAYKTF
SETSDYDDCVLPSTTETPVHDSKVTETKPFSLPPVAASSLKNNNDSS
```

Alligator sinensis: scaffold1076

Exon-intron structure:

Exon1	410510	410397
Exon2	393466	393404
Exon3	386288	386118
Exon4	380775	380613
Exon5	379189	379110

```
>Alligator_sinensis_cDNA
ACTTGGATTATCACTTGCTTTTATCTTCAGCTGCTCCTACTTAATCCACTTGTCAGAGCTCATAGTCCCTGTGGG
AATCCAGTGACAGATGATGTGAATGACATTACAATATTGGTTGGAAATCTTCCAAATGATTATAGGATAACCCTT
AAATATGTCGAGAAGATGGAAAGTCTGCCTAATCACTGTTGGTTGTATTTGATGGTGCCTGAATTTTCAAAGAGT
CTGAGTAATCTGCTCCACAAATTTTCACAAATATCTGATGTTTTAAGCAACCGTTCAATCATCAATAATCTCACA
AGGATAATTAATGATCTTATGGAATGTCTAGCATTAGATAAAAATAAGAATTTTAAACAAGAAAGCGATCACCTG
TATAAAGAAGGCAAGTTCATTCCTGAGGAATTCTTTAGGCATTTTAACAACACCATTGAAGCCTATAAGACGTTT
TCAGAAACATCAGATTATGATGACTGTGTTCTGCCTTCAACAACAGAAACACCAGTTCATGATTCCAAGGTCACT
GAAACAAAGCCATTTTCATTACCTCCTGTTGCTGCCAGTTCCCTTAAGAATAACAATGATAGCAGT

>Alligator_sinensis_Protein
TWIITCFYLQLLLLNPLVRAHSPCGNPVTDDVNDITILVGNLPNDYRITLKYVEKMESLPNHCWLYLMVPEFSKS
LSNLLHKFSQISDVLSNRSIINNLTRIINDLMECLALDKNKNFKQESDHLYKEGKFIPEEFFRHFNNTIEAYKTF
SETSDYDDCVLPSTTETPVHDSKVTETKPFSLPPVAASSLKNNNDSS
```

Crocodylus porosus: scaffold-17042

Exon-intron structure:

Exon1	21310	21423
Exon2	48730	48792
Exon3	55257	55427
Exon4	60678	60840
Exon5	64671	64750

```
>Crocodylus_porosus_cDNA
ACTTGGATTATCACTTGCTTTTATCTTCAGCTGCTCCTACTTAATCCACTTGTCAGAGCTCATAGACCCTGTGAG
AATCCAGTGACAGATGATGTGAATGACATTCCAAAATTGGTTGGAAATCTTCCAAATGATTATAGGATAACCCTT
AAATATGTCGAGAAGATGGAAAGTCTGCCTAATCATTGTTGGTTGCATTTGATGGTGCCTGAATTTTCAAAGAGT
CTGAATAATTTGCTCCACAAATTTTCACAAATATCTGAAGCTTTAAGCAACCACTCAATCATCAATAATCTCACA
AGGATAATTAATGATCTTATGGAATGTCTAGCATCAGATAAAAATAAGAATTTTAAAAAAGAAAGCGACCACCTC
TATAAAGAAGGCAAGTTCATTCCTGAGGAATTCTTTAGGCATTTTAACAACATCATTGAAGCCTTTAAGACATTT
TCAGAAATATCAGATTATGATGACTGTGTTCTGCCTTCAACAACAGAAACACCACTTAATGATTCCAAGGTCACT
GAAACAAAGCCATTTTCATTACCTCCTGTTGCTGCCAGTTCCCTTAAGAATAACAATGATAGCAGT

>Crocodylus_porosus_Protein
TWIITCFYLQLLLLNPLVRAHRPCENPVTDDVNDIPKLVGNLPNDYRITLKYVEKMESLPNHCWLHLMVPEFSKS
LNNLLHKFSQISEALSNHSIINNLTRIINDLMECLASDKNKNFKKESDHLYKEGKFIPEEFFRHFNNIIEAFKTF
SEISDYDDCVLPSTTETPLNDSKVTETKPFSLPPVAASSLKNNNDSS
```

Gavialis gangeticus: scaffold4318

Exon-intron structure

Exon1	102637	102524
Exon2	85583	85521
Exon3	66992	66822
Exon4	61638	61476
Exon5	60032	59953

```
>Gavialis_gangeticus_cDNA
ACTTGGATTATCACTTGCTTTTATCTTCAGCTGCTCCTACTTAATCCACTTGTCAGAGCTCAGCGTCGCTGTGAG
AATCCAGTGACAGATGATGTGAATGACATTCCAAAATTGGTTGGAAATCTTCCAAATGATTATAGGATAACCCTT
AAATATGTCGAGAAGATGGAAAGTCTGCCTAATCACTGTTGGTTGCATTTGATGGTGCCTGAATTTTCAAAGAGT
CTGAATAATTTGCTCCACAAATTTTCACAAATATCTGAAGCTTTAAGCAACCACTCAATCATCAATAATCTCACA
AGGATAATTAATGATCTTATAGAATGTCTAGCATCAGATAAAAATAAGAATTTTAAAGAAGAAAGCGACCACCTC
TATAAAGAAGACAAGTTCATTCCTGAGGAATTCTTTAGGCATTTTAACAACATCATTGAAGCCTTTAAGACATTT
TCAGAAATATCAGATTATGATGACTGTGTTCTGCCTTCAACAACAGAAACACCACTTAATGATTCTAAGGTCACT
GAAACAAAGCCATTTTCATTACCTCCTGTTGCTGCCAGCTCCCTTAAGAATAACAATGATAGCAGT

>Gavialis_gangeticus_Protein
TWIITCFYLQLLLLNPLVRAQRRCENPVTDDVNDIPKLVGNLPNDYRITLKYVEKMESLPNHCWLHLMVPEFSKS
LNNLLHKFSQISEALSNHSIINNLTRIINDLIECLASDKNKNFKEESDHLYKEDKFIPEEFFRHFNNIIEAFKTF
SEISDYDDCVLPSTTETPLNDSKVTETKPFSLPPVAASSLKNNNDSS
```

Nucleotide sequences of KIT-ligand retrocopies from CrocERV29

Sequences labeled “Cpor” are from *C. porosus*, “Ggan” are from *G. gangeticus*. The scaffold location is included within square brackets. Where the surrounding ERV insertions are relatively conserved, the name of the associated ERV sequence has also been provided.

>Cpor-scaffold-13420_[94543-93962];Crpo-13420-P00001
ATGATATCAACCACAAAATTGATCTGGATTGTCGCTCTCTTTGCGTTTGATCTACTCACTGCTGCTTCTCGCCCG
TGCGAAAATCCACTGACAGATGACGTCGATGACATCCCAAAATTGATTAAAAATCTTCCACACAATTATAAGATA
ACCCTTAAATATGTCAATAAAATGGAACATCTACACAATCATTGTTGGCTACATTTGATGGTGCCTCAATTCGCA
AAGAGCCTGAACAACTTGCTCCACAAATTTCCACAAACATTTGACGTGTCAAAAAACTACTCAATCCTCAACAGT
CTTACCAGAATGATTAACGATCTTCTAGAATGCCTCACATCAGACGGGCATAAATACAGCATTCATTCCTATAAA
GAAAGTGAATTCAGTCCTGAGGAATTCTTTAACCATTTCAATAATATGATTGAGGTCTTTAAGGCATTTTCAACA
ATACCGGCATATGACAATTGCGCCCCACGTTTTACGATGCAATATTCACAAGTCACCAAAACAAAGCTATCTTTT
CTACCTCCTGTCATTACTCCTTCCCTTCACAACGATACTGATAAAACT

>Cpor-scaffold-3177_[176589-176029];Crpo-3177_2-S00001
ATGACACCGACTGAGAATCTGACTTGGATTATTCTGCTCCTACTTAATCCACTTGTCAGAGCTCATAGACCGTGT
GAGAATCCAGTGACAGATGATGTGAATGATATCCCAAAATTGATTGAAAATCTTCCAAATGATTATAGGATAACC
CTTAAATATATCAAGAAGATGGAAAGTCTGCCTAATCATTGTTGGTTGCATTTGATGGTACCTGAATTTTCAAAG
AGTCTGAATAATTCGCTCCACAAATTTTCACAAATATCTGAAGCTTTAAGCAACCACTCAATCAGTAATAGTCTC
GCAAGGATAATGAACGATCTTATGGAATGTCTAGCATCAGATAAAAATAAGAACTTTAAAAGAGAAAGCAACCAC
CCCTATAAAGAAGGCGAGTTCATTCCTGAAGAATTCTTTAGGCATTTTAACAACATCGAGGCCTTTAAGATGTTT
TCAAATTATGACGACTGTGTTCTGCCTTCAACAACAGGAACACCACTTAATGATTCCAAAGTCACTGAAACAAAG
CCACTTTCATTGCAAGGAAGCCAGAGA

>Cpor-scaffold-5217_[4839-4289];Crpo-5217-S00001
ATGTCTACCATAAGACTTACTTGGGTTATTACTCTCTTTGCGCTTAATCTACTTCCCGCTACTTCTAATCCATGC
GGAAATCCAGTGACAGATGACGTCAAGGATATTCCAAAATTAATTAAAAATCTCCCAAGTGATTATAAGATAACC
CTCCAATATGTAAACCGATCGGAACTTCTACACAATCATTGCTGGCTACACTTGATGCTGCCTCACTTCGCTACA
AGTTTGAACAATTTACTCTATAAATTTCCACAAACATCTGACAACTACTTGATCCTCAACAGCCTCACCAGGATC
ATTCACGATATTCTAGAATGCCTCACGTCGGATGTATACGGTGTTCATTACGATGACCAATGTGACTTCAGTCCT
GAAGAATTCTTTGCCCACTTCAATCTTATACTTGAGTTCTTCAAGGCATCTTCAATGATACCGACATCTGATTGC
GTCCCGCTCCTTACGACACAGAATTTACAACTCACTGAAACAAAGCTACCTCCTGTTGTTACTCCTTCTGTCCTC
AACGATTATGATTA

>Cpor-scaffold-5863_[7253-7796];Crpo-5863-P00001
ATGATCATACAACTCACCTGGATCGTTACCTTCCTTACGCTCCGTCCACTTCCCGCTACTTCACATCCATGCGGA
AACTCCCTAATGGACGACGTTGAGGATATTCCGAAATTGATTGAAGGCCTTCCATGCGATTATACAATAACTCTC
CGATATGTGAATCAAACGGTACCTCTACATGATCATTGCTGGGTTCGCTTGATGATACCTCATTTTGCGACAAGC
CTGAGCAACCTGCTCTACAACTTTCCACCAATGTCTCATAACTATGTCATCCTCAATAGCCTCGCCAGGATGACC
CACAATCTTATGGAATGCCTCGCACCAGATACATACAATATTCATCCTTATGAAAAACATAAATACAGTCCTGAG
CGATATTTCGCCCACTTCAACGATATGGTTGACATCCTCAAAACGTCTTCAACGACACCGCCGAACAACTGCGCT
TCAGTCCTTACCACACATTTACAACTCGCCAAAACAGAGCTACCTCCTGTTGTTACTTCCTCTATTCTAAATAAT
TATG

>Ggan-scaffold-21115_[31040-30411];Gaga-21115-P00001
ATGACACCAACTAAAAATTGGATTTGGATTATCACTCTCTTCGTGCTTGATCTACTTACCGCTGCTTATAGGCAG
TGCGAGAATTCACTGACAGATGACGTGGATAACATCCCAGAATTGATTAAAAATCTTCCAACCGATTACAAAATA
ACCCTTAAATACATCAATGAGATGAGATATATCCACAAACATTGTTGGTTGCATTTGATGGTACCTGAATTTGCA
AAGAGCCTGAACAACTTGCTCCACAAATTTCCACGAACATCTAGTGCGTCAAACAACCACTCAATCCTCAACAGT
CTCACACGAGTGATTGACGATTTTCTAGAATGCCTCACGTCAGACCAACATGCGAACTTTAAAAAAGAAAGTGAC
CATTCCTATGCCGAAAGTGAATTCAGTCCTGAGGAATTCTTCAAGCAACTTAACAACATGATTGAAACCTTTAAG
GTGTTTTCAAAAATACTGGAGTATGATAACTGTGTTTTGTTTTCAACAACGGCAGCCCCGTTCACTGCCTCCGAG
GTCACTGGAACAGAACCACCTTATCCACCACCTCCTGTCGCCACCCGTTCCCTCAAAAAGGATTATCTACAAGAG
ACAGATGTAATGCTATCACCT

>Ggan-scaffold-8307_[28177-27575];Gaga-8307-S00001
ATGATACCGACCAAAAATTGGATTTGGATTATCACTCTTTTCGTGCTTGATCCACTTACCGCTGCTTATAGGCCG
TGCGAGAATCCACTGACAGATGACGTGGATGACATCCCAGAATTGATTAAAAATCTTCCAAACGATTATAAAATA

ACCCTTAAATATGTCAATGAGATGGGATATATCCACAAGCATTGTTGGCTGCATTTGATGGTACTTGAATTTGCA
AAGAGCCTGAACAACTTGCTCCACAAATTTCCACGAACATCTAGCGCGTCAAAAAACCACTCAATCCTCAACAGT
CTCACACGAATGGTTGACAAACTTCTAGAATACCTCACGTTGGATCAACAAAAGAACTTTAAAAAACAAAGTGAC
TATTCCTACGCAGAAAATGAATTCAGTCCTAAGGAATTCTTCAAACAACTTAACAACATGATTGAAACCTTTAAG
GTGTTTTCAAAAATACCGGAGTATGATAACTGCGTTTTCTCTTCAACAACGGAAGCCCTGTGTAATGCTTCCGAG
GTCACTGAAACAGAACTACCTTATCCACCACCTCCTGTCGCCACTCGTTCCCTCAAAAACGATTACCTA

>Ggan-scaffold-886_[32021-32683];Gaga-886-P00001
ATGACACCGACCAAAAACTTAATTTGGATTGTCACTTTCTTTACGCTTGATCTACTTACTGCTGCTTATAGACTG
TGCGAGAATTCACTGACAGATGACGTCGATAACATCCCAGAATTGATTAAAAATCTTCCGAGCGATTATAGGATA
ACCCTTAAATATGTCCATAAAGTGGAACACGTACACAATCATTGCTGGTTACATTTGATGGTACCCGAGTTTGCA
AAGAGCTTGAATAACTTACTCCACAAATTTCCAAGAACATCTGACGCCTCAGACAACCACTCAATCCTAAACAGC
CTCACCAGAATGATTAACGATCTTCTGGAATGCCTCACATCAGACAGATATAAAGACAGCGTCCATTCCTATCAA
GAAAGTGAATTCAGTCCCAAGGAATTCTTTAAACATTTCAATAATATGATTAAGATCTTTAAGGCATTTTCAACA
ATACCGGCGGATGATAATTGCGATCTGTTCTCCACAACGCCAACCCTGCTTAACGATTCAAAAGTCACTGATCCA
CCTCCTGTCGTTAAAAGTGCCCAAGATACTACCAGTTCTCACCTACAAACAATGAATGTCACGTCATCATCTACA
CCCAAAGACGAGAAGCTCGAACTTCCACCAGCTAGAAGAAAACCCGCTCAAGAG

>Ggan-scaffold-7393_[60286-59624];Gaga-7393-S00001
ATGACACTGACTAAAAACTTAATTTGGATTGTCACTCTCTTCATGCTTGATCTACTTACCGCTGCTTATAGACCG
TGCGAGAATTCACTGACCGATGACATCGATAACATCCCAGAATTGATTAAAAATCTTCCGAGAAATTATAGGATA
ACCCTTAAATATGTTCATAAAGTGGAACACATACACAATCATTGCTGGTTAGATTTGATGGTACCCAAGTTTGTA
AAGAGTTTGAATAGCTTACTCCACAAATTTCCAAAAACATCTGACGCCTCAGACAACCACTCAATCCTAAACAGC
CTCACCAGAATGATTAACGATCTTTGGGAATGCCTCACATCAGACGGATATAAAGACAGCATCCATTCCTATCAA
GAAAGTGAATTCAGTCCCGAGGAATTCTTTAAACATTTCAATAATATGATTAAGATCTTTAAGGCATTTTCAGCA
ATACCGGTGGATGATAATTGTGATCTGTTCTCCACGATGCAAACCCTGCTTAACGATTCAAAAGTCACTGATCCA
CCTCCTGTCGTTAAAAGTACCCAAGATACTACCAGTTCTCACCTACGAACGATGAATGTCACGTCATCATCTACA
CTGAAAAATGAGAAGCTCGAACTTCCACCAGCTGGAACAAAAACCACTCAAGAG

>Ggan-scaffold-7308_[209878-210558]
ATGACACTGACCGAGAGTCTAGTTTGGATTATCACGTGCTTTTATCTTCAGCTGCTCCTACTTAATCCGCTTGTC
AGAGCTCGTAAACCATGCGAGAATCCAGTGACAGATGATGTGAATGACATCCCAAAATTGATTAGGAATCTTCCA
AATGATTATAGGATAACCCTTAAATATGTCAAGAAGATGGAAAGTCTACCTAATCATTGTTGGTTGCATTTGATG
GTACCCAAATTTTCAAAGAGTCTAAACAATTTGCTCCACAAATTTTCACAGATATCTGAAGCTTTAAGCAACCAC
TCAATCATAAATAGTCTCACGAGGATAATTAATGATCTTATAGAATGTCTAGCAACAGATAAAAATAAGGACTTT
AAAAGAGAAAGCGACCACCCCTATGAAGAAGGCGAATTCATTCCTGAAGAATTTTTTTGGCATTTTAATAACATC
ATTAAAGCCTTTAAGATGTTTTCAGAGATATCGGATTATGATGACTATGTTCTGCCTCCAACAACAGAAACGAAG
CCACTTTCATTACCTCCTGTTACTGTCAGTTCCCTTAGGAAAAGCAATGATAGCAGTGTCCAGGACACTAGCAAC
TCCAGCCTGCAAGAGACCAGTGTAGCATGACCATCTAAACCAGAAAGTGAAGAACCTGAAGGGGGTAGTGAC

Protein sequences of KIT-ligand retrocopies from CrocERV29

>Cpor-scaffold-13420_[94543-93962];Crpo-13420-P00001
MISTTKLIWIVALFAFDLLTAASRPCENPLTDDVDDIPKLIKNLPHNYKITLKYVNKMEHLHNHCWLHLMVPQFA
KSLNNLLHKFPQTFDVSKNYSILNSLTRMINDLLECLTSDGHKYSIHSYKESEFSPEEFFNHFNNMIEVFKAFST
IPAYDNCAPRFTMQYSQVTKTKLSFLPPVITPSLHNDTDKT

>Cpor-scaffold-3177_[176589-176029];Crpo-3177_2-S00001
MTPTENLTWIILLLLNPLVRAHRPCENPVTDDVNDIPKLIENLPNDYRITLKYIKKMESLPNHCWLHLMVPEFSK
SLNNSLHKFSQISEALSNHSISNSLARIMNDLMECLASDKNKNFKRESNHPYKEGEFIPEEFFRHFNNIEAFKMF
SNYDDCVLPSTTGTPLNDSKVTETKPLSLQGSQR

>Cpor-scaffold-5217_[4839-4289];Crpo-5217-S00001
MSTIRLTWVITLFALNLLPATSNPCGNPVTDDVKDIPKLIKNLPSDYKITLQYVNRSELLHNHCWLHLMLPHFAT
SLNNLLYKFPQTSDNYLILNSLTRIIHDILECLTSDVYGVHYDDQCDFSPEEFFAHFNLILEFFKASSMIPTSDC
VPLLTTQNLQLTETKLPPVVTPSVLNDYD

>Cpor-scaffold-5863_[7253-7796];Crpo-5863-P00001
MIIQLTWIVTFLTLRPLPATSHPCGNSLMDDVEDIPKLIEGLPCDYTITLRYVNQTVPLHDHCWVRLMIPHFATS
LSNLLYNFPPMSHNYVILNSLARMTHNLMECLAPDTYNIHPYEKHKYSPERYFAHFNDMVDILKTSSTTPPNNCA
SVLTTHLQLAKTELPPVVTSSILNNY

>Ggan-scaffold-21115_[31040-30411];Gaga-21115-P00001
MTPTKNWIWIITLFVLDLLTAAYRQCENSLTDDVDNIPELIKNLPTDYKITLKYINEMRYIHKHCWLHLMVPEFA
KSLNNLLHKFPRTSSASNNHSILNSLTRVIDDFLECLTSDQHANFKKESDHSYAESEFSPEEFFKQLNNMIETFK
VFSKILEYDNCVLFSTTAAPFTASEVTGTEPPYPPPPVATRSLKKDYLQETDVMLSP

>Ggan-scaffold-8307_[28177-27575];Gaga-8307-S00001
MIPTKNWIWIITLFVLDPLTAAYRPCENPLTDDVDDIPELIKNLPNDYKITLKYVNEMGYIHKHCWLHLMVLEFA
KSLNNLLHKFPRTSSASKNHSILNSLTRMVDKLLEYLTLDQQKNFKKQSDYSYAENEFSPKEFFKQLNNMIETFK
VFSKIPEYDNCVFSSTTEALCNASEVTETELPYPPPPVATRSLKNDYL

>Ggan-scaffold-886_[32021-32683];Gaga-886-P00001
MTPTKNLIWIVTFFTLDLLTAAYRLCENSLTDDVDNIPELIKNLPSDYRITLKYVHKVEHVHNHCWLHLMVPEFA
KSLNNLLHKFPRTSDASDNHSILNSLTRMINDLLECLTSDRYKDSVHSYQESEFSPKEFFKHFNNMIKIFKAFST
IPADDNCDLFSTTPTLLNDSKVTDPPPVVKSAQDTTSSHLQTMNVTSSSTPKDEKLELPPARRKPAQE

>Ggan-scaffold-7393_[60286-59624];Gaga-7393-S00001
MTLTKNLIWIVTLFMLDLLTAAYRPCENSLTDDIDNIPELIKNLPRNYRITLKYVHKVEHIHNHCWLDLMVPKFV
KSLNSLLHKFPKTSDASDNHSILNSLTRMINDLWECLTSDGYKDSIHSYQESEFSPEEFFKHFNNMIKIFKAFSA
IPVDDNCDLFSTMQTLLNDSKVTDPPPVVKSTQDTTSSHLRTMNVTSSSTLKNEKLELPPAGTKTTQE

>Ggan-scaffold-7308_[209878-210558]
MTLTESLVWIITCFYLQLLLLNPLVRARKPCENPVTDDVNDIPKLIRNLPNDYRITLKYVKKMESLPNHCWLHLM
VPKFSKSLNNLLHKFSQISEALSNHSIINSLTRIINDLIECLATDKNKDFKRESDHPYEEGEFIPEEFFWHFNNI
IKAFKMFSEISDYDDYVLPPTTETKPLSLPPVTVSSLRKSNDSSVQDTSNSSLQETSVA
