# Supplementary material for: Evolution and gene capture in ancient endogenous retroviruses - insights from the crocodilian genomes
Source: Retrovirology. 2014 Dec 12;11:71. doi: 10.1186/s12977-014-0071-2 (PMC4299795; doi:10.1186/s12977-014-0071-2)
Supplement: Additional file 8: — Nectin3.pdf. Nectin3 genes from A. sinensis and nectin3-like sequences encoded by copies of CrocERV31. This file contains: Scaffold number, exon-intron locations, cDNA sequence, and amino acid sequence of the nectin3 gene from A. sinensis. Reconstructed cDNA and amino acid sequences of the nectin3-like retrocopies from CrocERV31 insertions. [file 12977_2014_71_MOESM8_ESM.pdf]

Additional File 8: Nectin3 genes from *A. sinensis* and nectin3-like sequences encoded by copies of CrocERV31.

This file contains:

- Scaffold number, exon-intron locations, cDNA sequence, and amino acid sequence of the nectin3 gene from *A. sinensis*.
- Reconstructed cDNA and amino acid sequences of the nectin3-like retrocopies from CrocERV31 insertions.

***A. sinensis* nectin3 gene**

Scaffold359

Exon-intron structure:

Exon1	957523	957185
Exon2	950794	950498
Exon3	947952	947835
Exon4	944216	944065
Exon5	927690	927101

```
>Alligator_sinensis_cDNA
GTGCCTTAGCTGGACCGATTGTTGATCCACACGTAACAGCAGTCTGGGGGAAGAATGTCACGTTAAAATGCATAA
TTGATATAAATGAAACAATAACACAAATCTCATGGGAGAAGATACATGGTAAAAGTGCACAAACTGTTGTTGTTC
ATCATCCTGAATATGGGTTTTCTGTTCAAAGAGAATACCATGGAAGAGTGTCATTTAAAAACTCTTCGCTCATTG
ATGCAACAATCAGCCTAAACAATGTGAGCTTCTCTGATGCAGGAGAGTACGTATGCAAAGCAGTTACATTCCCAC
TAGGAAATGCACAGTCGTCAACAACTGTAACTGTATTTGTTGAACCCACTGTGAGCCTTGCAAAGGGACCAAACT
CTTTAATAGATGGTGAAAATCAGACAGTAGCAGCCATTTGTACAGCAGCCACTGGGAAACCTGCTGCAGATGTTG
ATTGGGAAGGAGGCCTTGGTGAAATGGAATCTATTTCAACTTCCTTTCCAAATGAAACAGTGACAGTTGTAAGTC
AATATAAAATCATTCCTACCAGATTTGCAAGAGGAAGACGTATAACTTGTGTTGTAAAGCACCCAGCTTTGGAAA
AGGAGATAAGATATTCTTACGTGCTGGACATCCAATATGCCCCTGAAGTTTCAGTGACTGGCTATGATGGAAATT
GGTTCATTGGAAGAGGGAATGTTCAACTTAGGTGCAATGCTGATTCTAATCCACTACCTATGGAATTTACCTGGA
CCAGGCTGGATGGGCAATGGCCTGAAGGCTTACTGTCTATAAACAATACTCTGCATTTCAACAGCCCACTAACTT
ACAATTACACTGGGACTTACATCTGTAGGGTGACAAATTCCCTTGGTCAAAGAAGCGATCAAAAGACTATCTACA
TATTAGATCCTCCTACTACTACCACACGACCACCCACGGTTCCATGGCATCCTTCAACTGATGGCATCACAGGTT
TAGCAACAGAACCTAAACTAATATATTTTCCAACATCAACCTTGCCAGCAATGCAGGAAGACAATTGGGGTACCA
TCATCGGTAGTGCAGTGGGTGGGGCTCTCTTCCTGATACTTGTAAGCATTTTGGTTGGAGTTGTCTGCTATAGGA
GAAGACGGACGTTCCGTGGTGACTACTTTGCTAAGAACTACATTCCGCCAACTGATATGCAAAAAGAGTCTCAAA
TAGATGTTCTCCAGTCAGATGATGTGGATTCTTACCCTGACAGTGTAAAAAAAGAAATAAAGCATCCAATGAACA
GTCTGATATCTAAAGACTATTTAGAAGACCCTGAAAAGCCAGAGTGGAACAATGTAGGCAATATTAACAGGTACC
AGGAACGGTATGAAAGACCAATGGATTATTATGAAAGTGGAACACTGGGGATGAAATATATGGGTAGCGAATGTT
ATGATGACAATGAAGAAGACTTTGTTTCCCATGTAGATGGCTCAGTAATATCCCGAAGGGAGTGGTATGTG
```

```
>Alligator_sinensis_Protein
ALAGPIVDPHVTAVWGKNVTLKCIIDINETITQISWEKIHGKSAQTVVVHHPEYGFSVQREYHGRVSFKNSSLID
ATISLNNVSFSDAGEYVCKAVTFPLGNAQSSTTVTVFVEPTVSLAKGPNSLIDGENQTVAAICTAATGKPAADVD
WEGGLGEMESISTSFPNETVTVVSQYKIIPTRFARGRRITCVVKHPALEKEIRYSYVLDIQYAPEVSVTGYDGNW
FIGRGNVQLRCNADSNPLPMEFTWTRLDGQWPEGLLSINNTLHFNSPLTYNYTGTYICRVTNSLGQRSDQKTIYI
LDPPTTTTRPPTVPWHPSTDGITGLATEPKLIYFPTSTLPAMQEDNWGTIIGSAVGGALFLILVSILVGVVCYRR
RRTFRGDYFAKNYIPPTDMQKESQIDVLQSDDVDSYPDSVKKEIKHPMNSLISKDYLEDPEKPEWNNVGNINRYQ
ERYERPMDYYESGTLGMKYMGSECYDDNEEDFVSHVDGSVISRREWYV
```

Nucleotide sequences of nectin3 retrocopies from CrocERV31

All sequences were derived from *A. missisippiensis* ERV insertions, as this family appears to be species specific. Where the surrounding ERV insertions are relatively conserved, the name of the associated ERV sequence has also been provided.

```
>Amis-scaffold-8548_[AKHW01073359:7304-8593];Almi-JH736268-P00001
ATGGCTGCCGCAGGGGCACCGAGGCGGCGGACGTCCGCCGGGTTAGAGCTCGCTCTGGCACTGCTCCTTTCCCAT
TTCTGCTGTGGTACATTGATTAGACCGATTGTTGATCCACACGTAACAGCAGTTTGGGGAAAGAATGTCACATTA
AAATGCATAATTGATATAAAGGGAACAATAATACAAATTTCACAGGAAAAAATATATGGTAAAAGTGCACAAACT
GTTGTTGTATATCATCCTGAATATGGGTTTTCTTTTCAAAGAGAATACCATGGAAGAGTGTCATTTAAAAACTCT
TCACCCACTGATGCAACAATCAGACTAAACAATGTGAGCTTCTCTGATGCAGGAGAGTATGTATGTAAAATAGTT
ACATTCCCACTAGGAAATGCACAGTCGTCAACAACTGTAACTGTATTTGTTGAACCCACTGTGAGCTTTATAAAG
GGACCAAATTCTTTAATAGATGGTGAAAATCAGACAGTAGCAGCCATTTGTACAGCAGCCACTGGGAAACCTGCT
GCAGATATTGATTGGGAAGGAGACCTTGGCGAAATGGAGTTTAGTTTAACTTCCTTTCCAAATAAAATGGCGACA
GTTGTAAGTCAATATAAAATCATTCCTACCAGATTTGCCAGAGGAANACGTATAACTTGTGTCGTAAAGCACCCA
GCTTTGGACAAGGAAACAAGATACTCTTGCGTACTGGACATTCAATATGTCCCTGAAGTTTCAGCNACTGGCTAT
GACAGAAATTGGTTTATTGGAAGAGAGAATGTTCAGCTTAGGTGCAATGCTGCTTCTAATCCTCCACCTATNGAA
TTTGCCTGGACCAGGCTAGATAGGCAATGGCCTGAAGGCTTACTGTCTGTAAACAATACTCTGCATTTCAATAGC
CCACTAACCTACAATTACGCTGGGACTTACATCTGTAGGGTGACAAATTCCCTTGGTCAAGGAAGTGATCAAAAG
ACTATCCACATATTAGATCCTCTTATTACTATCACACGACCACCCACTGTTCCATGGCATCCTTCAACTAATAGC
ATCATAGGTTTAGCACCAGCATTAACCTTGCCAGCAATGCAGGAAGACAATCGGGGTACCATCATCGGTAGTGCG
GTTGGTGGAACCCTCTTCCTGGTATTTGCAAGTATTTTGGCTGGAGTTATCTGCTATGGGAGAAGACGAACGTTC
CGTGGTAACTATTTCGTTGAGAACTACATTCCGTCAACTGATGTAGAAAAGGAGTCCCAAATAGATGTTCCCCAG
TCAGATGATGACCAA

>Amis-scaffold-1726_[AKHW01016010:574-1860];Almi-JH732083_6-P00001
ATGGCCATGGAGAGGGCACCGAGGCAACAAATATCCGTCGGGTCAAAACTCGCTCTAATGCTGCTTCTCTCTCAT
TTCTGCTGCAGTGTATTAGTTAGACCTGTCGTTGATCCGTACGTAACAGCAGTTTGGGGAAAGAATGTCACATTA
AAGTGTATAATTAATATAAATGACACCATAATACAGGTTTCATGGGAAAAGATATATGATAAAAGTGCACAAACT
ATTGTTGTGCATTGTCCTGAATATGGGGTCTCTATTCAAAGAAAATACCATGGGAGGGTGTTATCCAGAAGTTCT
TTGCCCATTGATGCGACGATCAGTCTGCATAATGTGAGTTTCTATGATGAAGGAAAGTATAAATGCAAAGTAACT
ACATTCCCACTGGGAAATACACAATCATTAACAATTGTAACTGTACTTGTTAAGCCCACTGTAAGTTTCACCAAG
GAGCCAAATTCTCTAATAGATGATGGAAAGTGGACAGTGGCAGCTATCTGTACAGCAGCCACTGGAAAACCCGCT
GCNAATATAAGTTGGGAANGAGACTTTGGTAAAATAGAAAATAATTNAATTTCTTTTCCAAATAGAACAGTAACA
GTTATAAGCCNATATAAGATCATTCCCACTAAATTTGCGAGGGGAAGGCNTATAACTTGTATTGTGAAACACCCA
GCTTTGGACAGGGAAATAAGGTANTCTCACGTNCTAGACATCCAATATGCTCCTGAAGTNTCAATAACTGGNTAT
GACGGAAATTGGGTTTTTGGCAGAAAGAATGCTCAGCTTAATTGTAATGCTAATTCTAATCCGCCACCTCTAAAA
TTTACCTGGGCCAGATTAGATGGACAATGGCCTAAAGGCTTACTGTCTGTAAACAATACTCTGCATTTCAATAGC
CCACTAACTCACAGTCATAACGGAACTTATATTTGTAGGGTGACAAATTCCCTTGGTCAAAGAAGTAATCGAAAG
ACTATTTACGTATTAAATCTCTCCACCATCACTACGACACGACCACCCACCGTTTCATGGTATCCTTCAACTAAT
AGCATTACAGGTTTAGCGTCAGCATTAACCTCAACAGTAATTCAGGGACGCAGTTGGGGTACCATTATCAGTAGT
GCAGTAAATGTAAGTCTCTTTCTAGTACTGGGAAGTATTTTAATTGGCATTGTCTGCTATGGGAGAAGACGGACA
TTACGCAACGAGTATTTCGTTATGAACCGCATTTCACCAGCTAATAAGAGGGAGGAGTCCCCAACAGATGTTCTC
CAATCAGTTGATGACCCA

>Amis-scaffold-6002_[AKHW01052890:29546-28263]
ATGGCTGCCGTGGGGACACCAAGACTGCAAATGTTTGCTGGGTTAGAACTTCTTACGCTGCTTCTTTCCCATCTA
TGCTGTGGTGCTTTAGTCAAACCAATAGTTGATCCATACATAGCAGCAGTTTGGGGAAAGAATGTTACATTGAAA
TGTACAATCGACATGAATGAAACCATGATACAAATTTCTTGGGAAAAACTGCATGGTAAAAGTGCGGAAACTATT
GTCGTATATCATCCTGAATATGGGTTTTCTGTTCAAAGAGAATACCATGGAAGAGTGTCATTTAAAACCTCTTCG
TTCAGTGATATAACAATTGTCCTAAACAACGTGAGTTTCTCTGACGCAGGAAAGTATATATGCAAAGTAGTTACA
TTCCCATTAGGAAATGCAGAGTCATTAACAACTGTAACTGTAATTGCTGAACCCACTGTGAGCTTTACAAAGGAA
CCAACTCCCTTAATAGATAGTCAAAATTGGACAGTAGTAGCCATGTGTATAGCAGCCACAGGAAAACCTGCTGCA
AATATAAGTTGGGAAGGAGACTTTGGTAAAATGAAATTGAATTATATTTCCTTTCCAAATAGAACGGTAACAGTT
ATAAGCCAATATAAGATCATTCCCACTAAGTTTGCAACGGGAAGGCGTATAACTTGTATTGTGAAACACCCAGCT
TTGGACAGGGAAATAAGGTATTCTCACGTACTAGACATCCAATATGCTCCTGAAGTGTCAATAACTGGCTATAAT
AAGAATTGGTTTGTTGGACAAAAAAATGTTCAACTTAAGTGCAATACTAATTCTAACCCGCCGCCTATAAAGTTT
ACCTGGGCCAGACTAGATGGACAATGGCCTGAAGGCTTACTGTCGGTAAACAGTGCTTTGCATTTCAATAGCCCA
TTAACCCACAATCATGGCGGAACTTATATTTGTAGGGTGACAAATTCCCTTGGTCAAAGCAGCGATCAAAAGACT
GTTCACGTGTTAGATCTCTCTACCATCACTACTACACAATCATCCACGGTTCCACGGTATCCTTTAACTGATAAC
```

ATTGCGAGTTTGGCATCGGCCTTAACCTTAACAGCGATGCCGGAAAGCAATTGGGGTACCATTATCGGTAGTGTA
ACGGGTGCAGCTTTCTTTCTAATATTTGTAAGCATTTTAGTTGGAGTTATCTGTTACGGAAGAAGGCGCACGTTC
CGTGGCAGCTACGTCATTGAGGACCACACTCCGTCGACTGACGTAGAGGAAGAGTCTCAAGTAGACGTTTCCCAG
TCAACTGTT

>Amis-scaffold-11010_[AKHW01090924:1343-57]
ATGGAGGGGACATCGAGGCAGCGAATGTCCGCCGGGCCAGAACTTGTTCTGGCACTGCTCCTCCCTCATTTCTGC
TGCAGTGTGTTAGTTAGACCTATTGTTGATCCGTACGTAACAGCAGTTTGGGGAAAGAATGTCACATTAAAATGT
ATGATTGACAAGAATGAAACCATAATACAAGTTTTGTGGGAAAAGATATATGATAAAAATGCACAAACTATTGTT
GTGCATCATTCTGAATATGGGGTCTCTGTTCAAAGAAAATACCACGGGAGGGTGTTGTCTAGAAGTTCTTCGCCC
ACTGATGCGACAATCAGTATGTATAATGTGAGTTTCTATGATGAAGGAAAGTATATATGCAAAGTAACTACATTC
CCACTGGGAAATACACAATCGTTAACAATTGTAACTGTACTTGTTGAGCCCACTGTAAGTTTCAGCAAGGGGCCA
AATTCTCTAATAGATGATGGAAAGTGGGCACTGGCAGCCACTTGTACAGCAGCCATTGGGAAACCCGCTGCAAAT
ATAAGTCGGGAAGGAGACTTCGGTAAAATGGAATGTAATTCAATTTCCTTTCCAAATAGAACGGTAACAGTTATA
AGCCAATATAAGATCATTCCCACTAAATTTGCGAAGGGAAGGCGTATAACTTGTATTGTGAAACACCCATCTTTG
GACAGGGAAATAAGGTATTCTCACATACTAGACATCCAATATGCTCCTGAAGTGTCAATAACTGGCTATAATAAG
AATTGGTTTGTTGGACAAAGAAATGTTCAACTTAAGTGCAATGCTAATTCTAACCCGCCGCCTATAAAGTTTACC
TGGGCCAGACTAGATGGACAATGGTCTANAGGCTTACTGTCTGTAAACAGTGCTCTGCATTTCAACAGCCCATTA
ACTCACAATCATGGCGGGACTTACATTTGTAGGGTGATAAATTCCCTTGGTCAAAGAAGTGATCAAAAGACTATT
CACGTATTAGAGCTTTCTACCATCACTACCACACGATCACCCACGGTTTCATGGTATCCTTCAACTAATAGCATT
ACAGGTTTAGCATCAGCATTAACCTCAACAGTAATTCAGGGACGCAGCTGGGGTACCATCATCGGTAGTGCAGTA
AGTGTAAGTCTCTTTCTAGTACTGGGAAGTATTTTAATTGGCATTGTCTGCTACGGTAGAAAACGGATGTCTCGC
AGTAACTATTTTGTTATGAACCGCGTTTCACTGCCTGATAAAAGAGAGGAGTCCCAAACAGATGTTCTCCAATCA
GATGATGACCAA

>Amis-scaffold-5342_[AKHW01047614:11253-9973]
ATGGAGGGGACACCGAGGCAGCAAATGTCCGCCGGGCCAAAACTCGCTCTGGCACTGCTCCTCTCTCATTTCTGC
TGCAGTGTATTAGTTAGACCTGTTGTTGATCCGTATGTAACAGCAGTTTGGGGGAAGAATGTCACATTAAAATGT
ATGATTGACATGAATGAAACCATAATACAAGTTTCATGGGNAAAGATATATGATAAAAGTGCACAAACTATTGTT
GTGCATCATCCNGAATATGGGGTCTCTGTTCAAAGAAAATGCCATGGGAGGGTGTTGTCTAGAAGTTCTTCGCCC
ATTGATGCAACGATTAGTCTGCATAATGTGAGTTTCTATGATGAAGGAAAGTATATATGCAAAGTAACTACATTC
CCATTGGGAAATACACAATCGTTAACAATTGTAACTGTTCTTGTTGAGCCCACTGTAAGTTTTACCAAGGGGCCA
AATTCTCTAATAGATGATGGAAAGTGGGCAGTGGCAGCCATTTGTACAGCAGCCACTGGGAAACCCGCTGCAAAT
ATAAGCTGGGAAGGAGACTTTGGTAAAATGGAATATAATTCAATTTCCTTTCCAAATAGAACAGTAACAGTTATA
AGCCGATATAAGATCATTCTCACTAAATTTGCGAGGGGAAGGCGTATAACTTGTATTGTGAAACACCCAGCTTTA
AACAAAGAAATAAGATACTCTCACATACTAGACATCCAATATGCCCCTGAAGTATCAATAACTGGTTATGACGGA
AATTGGGTTTTCGGCGGAAAAATTGTTCAGCTTAAGTGCAAAGTTAATTCCAATCCACCACCTACAAAAGTCACC
TGGACCAGGCTAGATGAACAGTGGTCTGCGGGTTTAGTATCCGTGAGTGATATGCTATATTTCAGTGGCCCATTA
GCCTACGAATACATTGGAACCTGTATTTGTAGGGTAACAAATTCCATTGGTCAAAGAAGTGATCAACAAACTATC
CGTATATTATATCCTTCTATTATTACTACACAATCATCCACGGTTCCACGGTATCCTTTAACTAATAACATTACA
AGTTTGGCATCAGCCTTAACCTTAACAGCAATGCAGGAAGGCAATTGGGGTACCATTATCAGTAGTGTAATGGGT
GGAGCTTTCTTTCTAATATTTGTAAGCATTTTAGTTAGGGTTATCTGTTACAAAAGAAGGCGAACGTTCCGTGGC
AACTATGTCATTGAGGACCACACTCCGTCGACTGACATAGAGGAAGAGTTTCAAGTAGATGTTTCCCAGTCAGCT
GATGAT

>Amis-scaffold-9090_[AKHW01077532:3696-2134]
ATGGAAAACACACATTGGCCCTTTCCACTCCCGGCGCTTGTGGTGGGACAAGTGACACACGCACCTGGCGTGGGG
AGACGGCGGCGGCGTTGGCAGGTGCCCGCCGGGCTGGGGCTCGCTCTGGCGCTGCTCCTCTTCCGCTTCTGCTGC
ACTGCCTTAGCTGGACTGATTGTTGATCCACACGTAACAGCAGTCTGGGGGAAGAATGTCACGTTAAAATACATA
ATTGTTATAAATGAAACAATAACACAAATCTCATGGGAGAAGATACATGGTAAAAGTGCACAAACTGTTGTTGTA
CATCATCCTGTATATGGGTTTTCTGTTCAAAGAGAATACCATGGAAGAGTGTCATTTAAAAACTCTTCGCTCATT
GATGCAACAATCAGCCTAAACAATGTGAGCTTCTCTGATGCAGGAGAGTATGTATGCAAAGCAGTTATATTCCCA
CTAGGAAATGCACAGTCGTCAACAACTGTAACTGTATTTGTTGAACCCATTGTGAGCCTTACAAAGGGACCAAAC
TCTTTAATAGATGGTGAAAATCAGACAGTAGCAGCCATTTGTACAGCAGCCACTGGGAAACCTGCTGCAGATGTT
GATTGGGAAGGANGNCTTGGTGAAATGGAATCTAGTTCAACTTCCTTTCCAAATGAAACAGTGACAGTTGTAAGT
CAATATAAAATCATTCCTACCAGATTTGCAAGAGGAAGACATATAACTTGTGTTGTAAAGCACCCAGCTTTGGAA
AAGGAGATAAGATATTCTTACGTGCTGGACATCCAATATGCCCCTGAAGTTTCAGTGACTGGCTATGATGGAAAT
TGGTTCATTGGAAGAGAGAATGTTCAGCTTAGGTGCAATGCTGATTCTAATCCACTACCTATGGAATTCACCTGG
ACCAGGCTGGATGGGCAATGGCCTGAAGGCTTACTGTCTATAAACCATACTCTGCATTTCAACAGCCCACTAACC
TACAATTACACTGGGACTTGCATCTGTAGGGTGACAAATTCCCTTGGTCAAAGAAGCGAACAAAAGACTATCTAC
ATATTAGATCCTCCTACTACTACCACACGACCATCCACAGTTCCATGGCATCCTTCAACTGATGGCATCACAGGT

TTAGCAACAAAACCTAAACTAATATATTTTCCAGCATCAACCTTGCCAGTAATGCAGGAAGACAATTGGGGTACC
ATCATCGGTAGTGCAGTGGGTGGGGCTCTCTTCCTGATACTTGTAAGCATTTTGGTTGGAGTTGTCTGCTATAGG
AGAAGATGGACATTCTGTGGTGACTACTTTGCTAAGAACTACATTCCGCCAACTGATATACAAAAAGAGTTTCAA
ATAGATGTTCTCCAGTCAGATGATGTGGATTCTTACCCTGACAGTGAAAACAAAAAAGAAATAAAGCATCCAATG
AAAAGTCTGATATCTAAAGACTATTTAGAAGACCCTGAAAAGCCAGAGTGGAACAATGTAGGCAATATTAACAGG
TACCAGGAACGGTATGAAAGACCAGTGAACTGTTGTGTTTCTTTAAGGTACTGCAACACTGTC

Protein sequences of nectin3 retrocopies from CrocERV31

>Amis-scaffold-8548_[AKHW01073359:7304-8593];Almi-JH736268-P00001
MAAAGAPRRRTSAGLELALALLLSHFCCGTLIRPIVDPHVTAVWGKNVTLKCIIDIKGTIIQISQEKIYGKSAQT
VVVYHPEYGFSFQREYHGRVSFKNSSPTDATIRLNNVSFSDAGEYVCKIVTFPLGNAQSSTTVTVFVEPTVSFIK
GPNSLIDGENQTVAAICTAATGKPAADIDWEGDLGEMEFSLTSFPNKMATVVSQYKIIPTRFARGXRITCVVKHP
ALDKETRYSCVLDIQYVPEVSATGYDRNWFIGRENVQLRCNAASNPPPXEFAWTRLDRQWPEGLLSVNNTLHFNS
PLTYNYAGTYICRVTNSLGQGSDQKTIHILDPLITITRPPTVPWHPSTNSIIGLAPALTLPAMQEDNRGTIIGSA
VGGTLFLVFASILAGVICYGRRRTFRGNYFVENYIPSTDVEKESQIDVPQSDDDQ

>Amis-scaffold-1726_[AKHW01016010:574-1860];Almi-JH732083_6-P00001
MAMERAPRQQISVGSKLALMLLLSHFCCSVLVRPVVDPYVTAVWGKNVTLKCIININDTIIQVSWEKIYDKSAQT
IVVHCPEYGVSIQRKYHGRVLSRSSLPIDATISLHNVSFYDEGKYKCKVTTFPLGNTQSLTIVTVLVKPTVSFTK
EPNSLIDDGKWTVAAICTAATGKPAANISWEXDFGKIENNXISFPNRTVTVISXYKIIPTKFARGRXITCIVKHP
ALDREIRXSHVLDIQYAPEVSITGYDGNWVFGRKNAQLNCNANSNPPPLKFTWARLDGQWPKGLLSVNNTLHFNS
PLTHSHNGTYICRVTNSLGQRSNRKTIYVLNLSTITTTRPPTVSWYPSTNSITGLASALTSTVIQGRSWGTIISS
AVNVSLFLVLGSILIGIVCYGRRRTLRNEYFVMNRISPANKREESPTDVLQSVDDP

>Amis-scaffold-6002_[AKHW01052890:29546-28263]
MAAVGTPRLQMFAGLELLTLLLSHLCCGALVKPIVDPYIAAVWGKNVTLKCTIDMNETMIQISWEKLHGKSAETI
VVYHPEYGFSVQREYHGRVSFKTSSFSDITIVLNNVSFSDAGKYICKVVTFPLGNAESLTTVTVIAEPTVSFTKE
PTPLIDSQNWTVVAMCIAATGKPAANISWEGDFGKMKLNYISFPNRTVTVISQYKIIPTKFATGRRITCIVKHPA
LDREIRYSHVLDIQYAPEVSITGYNKNWFVGQKNVQLKCNTNSNPPPIKFTWARLDGQWPEGLLSVNSALHFNSP
LTHNHGGTYICRVTNSLGQSSDQKTVHVLDLSTITTTQSSTVPRYPLTDNIASLASALTLTAMPESNWGTIIGSV
TGAAFFLIFVSILVGVICYGRRRTFRGSYVIEDHTPSTDVEEESQVDVSQSTV

>Amis-scaffold-11010_[AKHW01090924:1343-57]
MEGTSRQRMSAGPELVLALLLPHFCCSVLVRPIVDPYVTAVWGKNVTLKCMIDKNETIIQVLWEKIYDKNAQTIV
VHHSEYGVSVQRKYHGRVLSRSSSPTDATISMYNVSFYDEGKYICKVTTFPLGNTQSLTIVTVLVEPTVSFSKGP
NSLIDDGKWALAATCTAAIGKPAANISREGDFGKMECNSISFPNRTVTVISQYKIIPTKFAKGRRITCIVKHPSL
DREIRYSHILDIQYAPEVSITGYNKNWFVGQRNVQLKCNANSNPPPIKFTWARLDGQWSXGLLSVNSALHFNSPL
THNHGGTYICRVINSLGQRSDQKTIHVLELSTITTTRSPTVSWYPSTNSITGLASALTSTVIQGRSWGTIIGSAV
SVSLFLVLGSILIGIVCYGRKRMSRSNYFVMNRVSLPDKREESQTDVLQSDDDQ

>Amis-scaffold-5342_[AKHW01047614:11253-9973]
MEGTPRQQMSAGPKLALALLLSHFCCSVLVRPVVDPYVTAVWGKNVTLKCMIDMNETIIQVSWXKIYDKSAQTIV
VHHPEYGVSVQRKCHGRVLSRSSSPIDATISLHNVSFYDEGKYICKVTTFPLGNTQSLTIVTVLVEPTVSFTKGP
NSLIDDGKWAVAAICTAATGKPAANISWEGDFGKMEYNSISFPNRTVTVISRYKIILTKFARGRRITCIVKHPAL
NKEIRYSHILDIQYAPEVSITGYDGNWVFGGKIVQLKCKVNSNPPPTKVTWTRLDEQWSAGLVSVSDMLYFSGPL
AYEYIGTCICRVTNSIGQRSDQQTIRILYPSIITTQSSTVPRYPLTNNITSLASALTLTAMQEGNWGTIISSVMG
GAFFLIFVSILVRVICYKRRRTFRGNYVIEDHTPSTDIEEEFQVDVSQSADD

>Amis-scaffold-9090_[AKHW01077532:3696-2134]
MENTHWPFPLPALVVGQVTHAPGVGRRRRRWQVPAGLGLALALLLFRFCCTALAGLIVDPHVTAVWGKNVTLKYI
IVINETITQISWEKIHGKSAQTVVVHHPVYGFSVQREYHGRVSFKNSSLIDATISLNNVSFSDAGEYVCKAVIFP
LGNAQSSTTVTVFVEPIVSLTKGPNSLIDGENQTVAAICTAATGKPAADVDWEGXLGEMESSSTSFPNETVTVVS
QYKIIPTRFARGRHITCVVKHPALEKEIRYSYVLDIQYAPEVSVTGYDGNWFIGRENVQLRCNADSNPLPMEFTW
TRLDGQWPEGLLSINHTLHFNSPLTYNYTGTCICRVTNSLGQRSEQKTIYILDPPTTTTRPSTVPWHPSTDGITG
LATKPKLIYFPASTLPVMQEDNWGTIIGSAVGGALFLILVSILVGVVCYRRRWTFCGDYFAKNYIPPTDIQKEFQ
IDVLQSDDVDSYPDSENKKEIKHPMKSLISKDYLEDPEKPEWNNVGNINRYQERYERPVNCCVSLRYCNTV
